# Multiple weak interactions between BvgA~P and *ptx* promoter DNA strongly activate transcription of pertussis toxin genes in *Bordetella pertussis*

**DOI:** 10.1371/journal.ppat.1008500

**Published:** 2020-05-13

**Authors:** Qing Chen, Philip E. Boucher, Scott Stibitz

**Affiliations:** Division of Bacterial, Parasitic, and Allergenic Products, Center For Biologics Evaluation and Research, FDA, Silver Spring, Maryland, United States of America; University of California, Davis, UNITED STATES

## Abstract

Pertussis toxin is the preeminent virulence factor and major protective antigen produced by *Bordetella pertussis*, the human respiratory pathogen and etiologic agent of whooping cough. Genes for its synthesis and export are encoded by the 12 kb *ptx-ptl* operon, which is under the control of the pertussis promoter, P*ptx*. Expression of this operon, like that of all other known protein virulence factors, is regulated by the BvgAS two-component global regulatory system. Although P*ptx* has been studied for years, characterization of its promoter architecture vis-à-vis BvgA-binding has lagged behind that of other promoters, mainly due to its lower affinity for BvgA~P. Here we take advantage of a mutant BvgA protein (Δ127–129), which enhances *ptx* transcription in *B*. *pertussis* and also demonstrates enhanced binding affinity to P*ptx*. By using this mutant protein labeled with FeBABE, binding of six head-to-head dimers of BvgA~P was observed, with a spacing of 22 bp, revealing a binding geometry similar to that of other BvgA-activated promoters carrying at least one strong binding site. All of these six BvgA-binding sites lack sequence features associated with strong binding. A genetic analysis indicated the degree to which each contributes to P*ptx* activity. Thus the weak/medium binding affinity of P*ptx* revealed in this study explains its lower responsiveness to phosphorylated BvgA, relative to other promoters containing a high affinity binding site, such as that of the *fha* operon.

## Author summary

The functional architecture of the promoter directing pertussis toxin production, P*ptx*, in *Bordetella pertussis* remains largely unknown due to its weaker binding to the activator BvgA~P. In this study we used a mutant BvgA possessing increased binding affinity to P*ptx*, relative to wild-type BvgA. Using this mutant protein in FeBABE-labeled BvgA footprinting, we have obtained a base-pair resolution picture of six dimers of BvgA~P binding to P*ptx* and have revealed a binding geometry similar to other BvgA-activated promoters carrying a high affinity binding site. However P*ptx* lacks any strong binding sites. The requirement of multiple weak/medium binding affinity sites for the activation of P*ptx*, demonstrated in this study, provides a mechanistic framework for understanding the pattern of late gene expression observed for pertussis toxin, relative to early gene promoters containing strong binding sites.

## Introduction

Like many bacterial pathogens, *Bordetella pertussis*, the causative agent of the human disease whooping cough, controls the expression of multiple virulence genes via a central regulatory locus. In *B*. *pertussis*, this is the *bvgASR* locus, comprising the *bvgAS* operon, which encodes the two-component system BvgAS, and the convergently transcribed *bvgR* gene that mediates transcriptional suppression of Bvg-repressed genes, or *vrg*s (*vir* repressed genes). The BvgAS two-component system is somewhat atypical in that, in the absence of specific stimuli, the sensor kinase BvgS actively phosphorylates the response regulator BvgA. This is called the Bvg^+^ mode. The reciprocal state, the Bvg^-^ mode, is induced by exposure to environmental signals. In the case of BvgAS these signals are specific compounds, termed modulators, typified by MgSO_4_ and nicotinic acid, or low temperatures (26°C).

Phosphorylated BvgA (BvgA~P) activates the promoters of virulence genes, or *vag*s (*vir* activated genes), by binding to upstream target sites, and is both necessary and sufficient in this regard. Transcription of multiple virulence gene promoters has been demonstrated to be activated by BvgA~P alone *in vitro*. Included are those driving expression of: *fha* [1–4], *ptx* [1], *cya* [1], *prn* [5], *bipA* [6], and *fim3* [4, 7]. However the architecture of these promoters varies in terms of number, apparent affinity, and placement, of BvgA-binding sites relative to the -35 and -10 core promoter elements. Some promoters, typified by those driving expression of genes encoding putative adhesins, have a smaller number of higher affinity binding sites. These include the promoters of the *fha* operon encoding filamentous hemagglutinin, the *bipA* gene encoding an outer membrane protein, the *fim2*, *fim3*, and *fimX* genes encoding fimbrial subunits of different serotypes, and the *prn* gene encoding the outer membrane adhesin pertactin. In the case of the promoters P*ptx* and P*cya*, which drive production of the two most-studied protein toxins, pertussis toxin and adenylate cyclase toxin, the BvgA-binding region is larger and apparently of lower affinity. The effects of these differences in promoter architecture *in vivo* has been revealed in multiple ways. Scarlato et al. [8] demonstrated that, after shifting a *B*. *pertussis* culture from Bvg^-^ to Bvg^+^ conditions, transcription of *fha* genes was detected within minutes, while that of *ptx* and *cya* did not begin for several hours. This was interpreted as differential responsiveness of these promoters to rising intracellular concentrations of BvgA following the shift. While these authors did demonstrate increasing levels of BvgA protein, only more recently was it confirmed that the levels of BvgA~P rise concomitantly under these conditions [9]. Differential responsiveness of promoters can also be revealed by “modulation curves” whereby steady state cultures in differing concentrations of a modulator, such as MgSO_4_, are examined for virulence gene expression [10]. Also, using the RIVET approach, it was shown that the dynamic succession of gene transcription following removal of modulating signals, could also be observed *in vivo*, in a mouse model of infection [11].

Analysis of the *fhaB* promoter:BvgA~P:RNAP ternary transcription initiation complex *in vitro* has provided a more detailed picture of its architecture. Assembling functional complexes in which either BvgA or the alpha subunit of RNA polymerase was labeled with the conditional cleavage moiety Fe-BABE allowed a determination of the number, orientation, and location of BvgA monomers and also revealed a novel mode of interaction of the alpha subunit C-terminal (α-CTD) domain within a ternary complex [12]. Since demonstrated at additional Bvg-regulated promoters [13], in this configuration the α-CTD binds to the same linear segment of the promoter DNA as BvgA~P, but to a different helical face. BvgA~P was observed to bind as head-to-head dimers of the BvgA monomer, with the centers of binding spaced at 22 bp, or two helical turns of the DNA (one monomer per helical turn). Thus all BvgA dimers appear to be bound to the same face of the DNA helix, presumably stabilized by intra-dimer interactions. It should be noted that for strong binding sites, such as the primary binding site of P*fha*, dimer binding is centered on an inverted heptad that represents an optimal BvgA-binding site [14]. However, DNA-sequenced-based prediction of the presence and location of lower affinity sites is problematic, as illustrated in this study. Here we have characterized P*ptx* lower affinity binding sites by first observing binding of BvgA~P labeled with iron bromoacetamidobenzyl-EDTA (FeBABE). Binding strength of the corresponding DNA sequence can then be inferred using an algorithm based on a systematic mutagenesis study of the P*fha* high affinity primary binding site [14]. As previously shown for P*fha*, and shown here for P*ptx*, at the downstream end of the BvgA-binding region, the most promoter-proximal binding site abuts the promoter’s -35 region. Taken together, this binding site architecture is consistent with productive contacts between BvgA and the sigma and alpha subunits of RNAP.

The subject of this study, the *ptx* promoter, contrasts with the *fhaB* promoter in a number of ways. Firstly, as described above, this promoter is less responsive to BvgA~P, requiring higher levels for activation [9]. Secondly, based on genetic mapping and DNase footprinting, P*ptx* appears to contain a larger BvgA-binding region does than P*fha* [15,16], although the linear spatial resolution of the DNase footprinting techniques that have been applied to date have not allowed a precise determination of the number or location of BvgA molecules bound to P*ptx*. Finally, although a consensus, high-affinity BvgA-binding site is present in P*fha*, no obvious matches to this consensus are found within the BvgA-binding region of P*ptx*. This, together with the requirement for higher BvgA~P concentrations to observe binding to P*ptx in vitro*, indicates that BvgA binding sites within P*ptx* are of lower affinity. In order to understand how, in spite of these factors, P*ptx* is able, at the levels of BvgA~P encountered in the Bvg^+^ mode, to promote transcription at a level comparable to that of P*fha*, we undertook a systematic study of its structure and function.

## Results

### Isolation of a BvgA mutant with increased activation of P*ptx*

**Fig 1:**
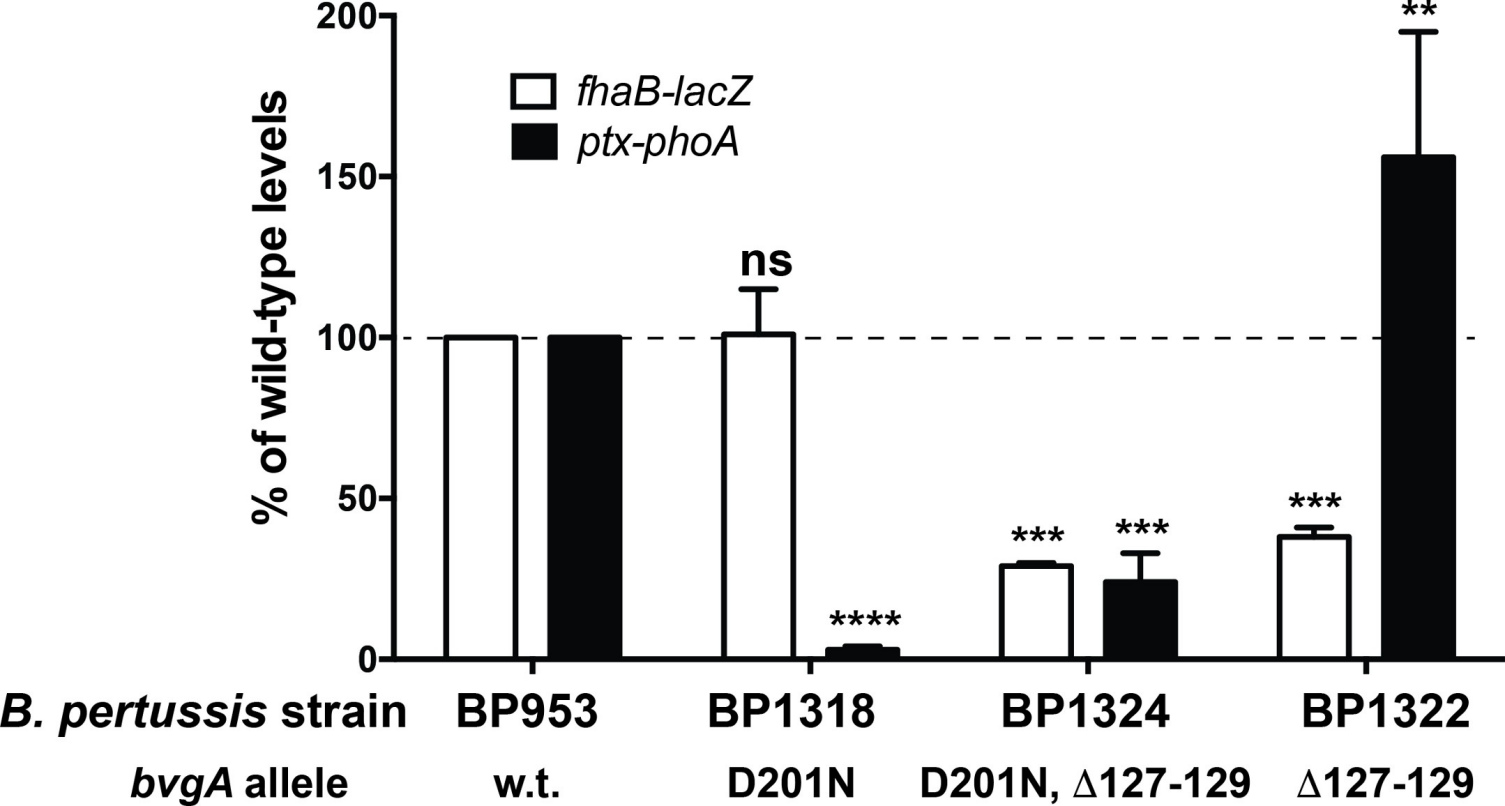
Activity of gene fusions in BP953 and derivatives. BP953 contains two transcriptional fusions, *fha-lacZ* and *ptx-phoA*. The results of beta-galactosidase and alkaline phosphatase enzymatic assays are shown, with w.t. values set to 100% for comparison. Also shown are the results of assays of the derivatives, BP1318, BP1324, and BP1322 harboring the *bvgA* alleles indicated, normalized to those for BP953. Isolation of these alleles is described in the text. Values were normalized to *fha-lacZ* and *ptx-phoA* expressed in BP953 and data from at least four assays were used in the calculation of means, standard deviations, as indicated by error bars, and statistical analysis by one-way ANOVA. Outcomes of the latter analysis are presented using the symbols: ns, P > 0.05; **, P ≤ 0.01; ***, P ≤ 0.001; ****, P ≤ 0.0001.

Previously, prior to the demonstration that BvgA~P was both necessary and sufficient to activate *ptx* transcription, we reported the isolation of mutant *B*. *pertussis* strains displaying a phenotype consistent with a defect in a hypothetical *ptx*-specific transcriptional activator [17]. The existence of such a regulator had been invoked to explain how the *bvgAS* locus could activate P*fha*, but not P*ptx*, in *E*. *coli*, and why BvgA could be demonstrated to bind to P*fha*, but not to P*ptx*, *in vitro*. These mutants were isolated as Lac^+^ Pho^-^ variants following chemical mutagenesis of the *B*. *pertussis* strain BP953, harboring *fha-lacZ* and *ptx-phoA* transcriptional fusions. BP1056 was one of these strains, in which *ptx-phoA* expression was highly deficient relative to wild-type, but *fha-lacZ* expression was normal. The mutation responsible for this phenotype was genetically mapped to the *bvgA* gene and sequence analysis revealed that it resulted in the amino acid substitution D201N [17]. In order to isolate suppressor mutations of *bvgA*^D201N^, by selecting for strains in which *ptx* expression was restored, a promoterless kanamycin resistance gene was added in-line with the *phoA* gene of BP1056 as described in Materials and Methods. Subsequent selection of spontaneous mutants surviving selection for kanamycin resistance and exhibiting increased alkaline phosphatase activity were obtained and analyzed further. One of the candidates characterized in more detail was BP1286, Using the previously described method of allelic retrieval and subsequent allelic exchange [18], we transferred the *bvgA* gene of BP1286 into *B*. *pertussis* BP953, thereby creating BP1324. This was done to eliminate the possible contribution of background mutations and to verify that the suppressor phenotype was encoded within the *bvgA* gene itself. Using this approach, BP1318 and BP1324 were created as “clean” versions of BP1056 and BP1286, containing the *bvgA*^D201N^ and *bvgA*^D201N, Δ127–129^ alleles, respectively. Sequence analysis of the *bvgA* gene in BP1324 indicated that the suppressor mutation was a deletion of 9 bp in the *bvgA*^D201N^ gene, resulting in the deletion of amino acids 127 to 129 (STT) of the BvgA D201N protein (*bvgA*^D201N, Δ127–129^). To create a strain in which only the Δ127–129 mutation was present, the allelic exchange plasmid pSS2429, derived from BP1286, and containing both mutations was used in “fragment swapping” cloning to replace the D201N mutation with its wild-type counterpart. The resulting plasmid pSS2427 was used to introduce just the Δ127–129 deletion into BP953 to create BP1322. As shown in Fig 1, BP1324 manifested the Lac^+^ Pho^+^ phenotype, with quantitative enzyme assays revealing a significantly higher level of *ptx* transcription, and unexpectedly a somewhat decreased level of *fha* transcription. When strain BP1322, harboring the *bvgA*^Δ127–129^ mutation in the absence of the original *bvgA*^D201N^ mutation, was examined, it was observed that levels of *ptx* transcription were even higher than wild-type levels, while *fha* transcription remained somewhat lower than the wild-type.

### BvgA^Δ127–129^ is not more highly phosphorylated *in vivo*

**Fig 2:**
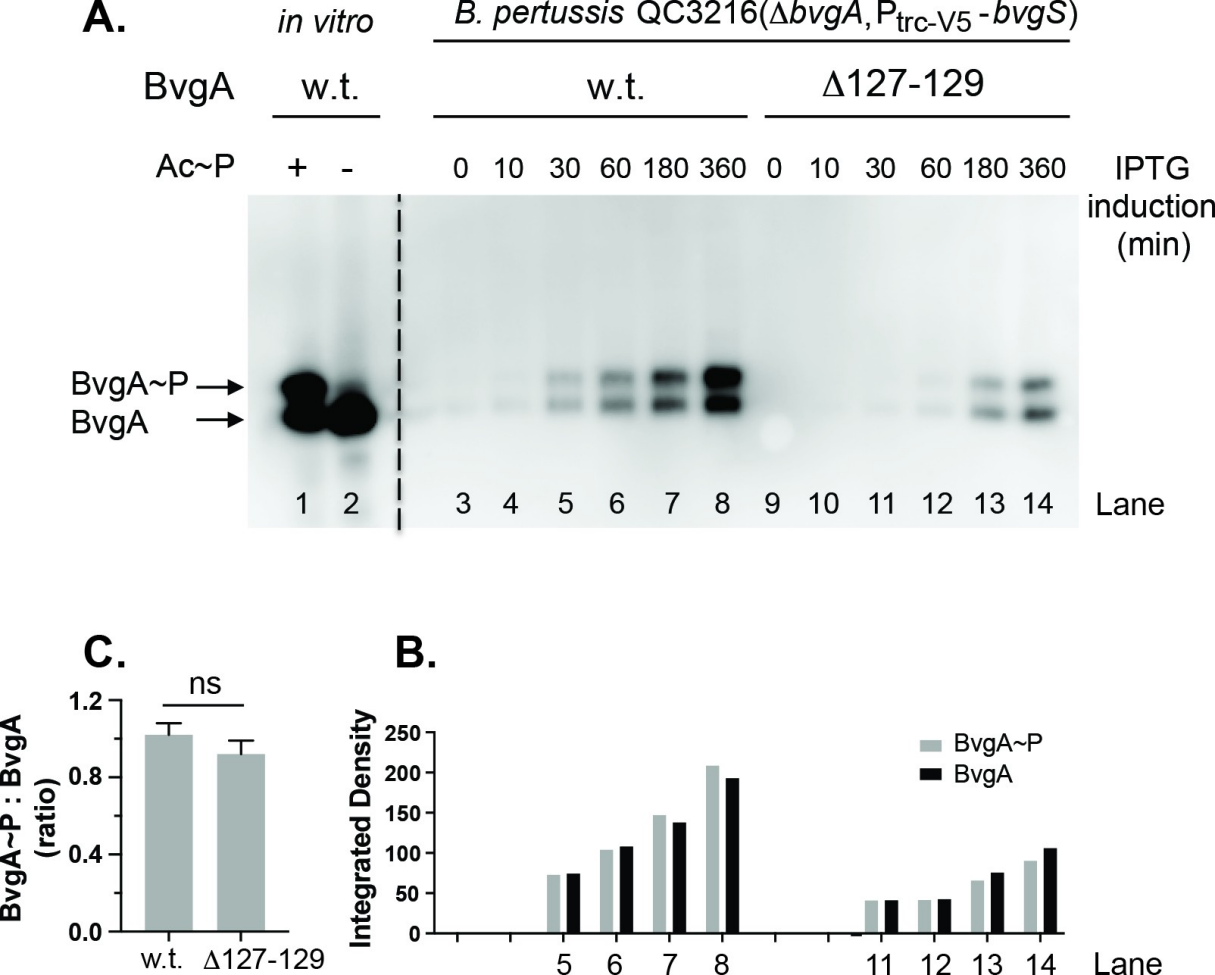
Kinetics of *in vivo* phosphorylation of wild type and Δ127–129 BvgA. **A.** *B*. *pertussis* strain QC3216 harboring plasmids conferring expression of wild-type BvgA (pSS4893, lanes 3–8) and BvgA^Δ127–129^ (pQC1894, lane 9–14), respectively, were grown in PLB liquid media, induced with 1 mM IPTG and sampled at various time points post induction. The collected samples were analyzed by Phos-tag gel electrophoresis, followed by Western blot with anti-BvgA detection, as previously described [9]. Control lanes contained 1 pmol of purified BvgA incubated in the presence (+, lane 1) or absence (-, lane 2) of acetyl phosphate as described previously [9]. **B.** The intensities of BvgA (black bar) and BvgA~P (grey bar) for lanes 5–8 and 11–14 were quantified and reported as integrated density using ImageJ software. **C.** The quantitative intensities derived from four IPTG-induction time points (30 min, 60 min, 180 min and 360 min) in panel B were used to calculate the ratios of BvgA~P to BvgA for the wild type and the mutant BvgA, respectively, and to obtain the means, standard deviations, as indicated by error bars, and statistical analysis by one-way ANOVA. Outcome of the latter analysis is presented using the symbol: ns, P > 0.05.

To understand why BP1322 (*bvgA*^Δ127–129^) displayed increased activation of P*ptx*, we first assessed whether the Δ127–129 mutation affected BvgA phosphorylation. To allow expression in *B*. *pertussis*, the *bvgA*^Δ127–129^ gene was cloned into the IPTG-inducible *lac* promoter-based expression vector pQC1883, capable of replication in *B*. *pertussis* [9], to create pQC1894. A version of this plasmid containing the wild-type *bvgA* gene, pSS4983, has previously been described [9]. The *B*. *pertussis* strain QC3216 (BP536, Δ*bvgA*, P2-*bvgS*) was used as a host for both plasmids in these experiments. This strain contains an in-frame deletion of *bvgA*, with a constitutive P*trc* derivative driving expression of *bvgS* [9]. Cultures of the strain QC3216 harboring plasmids pSS4983 and pQC1894, respectively, in PLB liquid media, were induced with 1 mM IPTG and samples were collected at various times post-induction. Expression and phosphorylation of plasmid-encoded wild-type BvgA or BvgA^Δ127–129^ proteins were assessed by Phos-Tag gel electrophoresis, followed by Western blot probed with an anti-BvgA monoclonal antibody. As shown in Fig 2A, both phosphorylated BvgA (BvgA~P) and unphosphorylated BvgA (BvgA) were detected *in vivo* for the plasmid-encoded wild-type BvgA (lanes 3–8) and BvgA^Δ127–129^ protein (lanes 9–14) in strain QC3216 after extended IPTG induction. Purified wild type BvgA incubated in *vitro* with or without phosphate donor acetyl phosphate (Ac~P, Fig 2A lanes 1&2) was used as a control, as described previously [9]. We observed lower expression levels of the BvgA^Δ127–129^ protein than those of the wild-type (Fig 2B). Since both the wild type and the mutant BvgA were expressed from the same plasmid vector and in the same genetic context where BvgS levels were constant and not affected by BvgA-dependent auto-regulation, the difference in expression is likely due to differences in translation efficiency or protein stability. Nevertheless, the ratios of phosphorylated BvgA protein to the unphosphorylated BvgA in both the wild type and the mutant BvgA were similar (Fig 2C). These results allow us to conclude that the BvgA^Δ127–129^ protein is not more highly phosphorylated *in vivo*.

### Phosphorylated BvgA^Δ127–129^ binds P*ptx* with higher affinity and provides more extensive protection in DNase I footprinting assays

**Fig 3:**
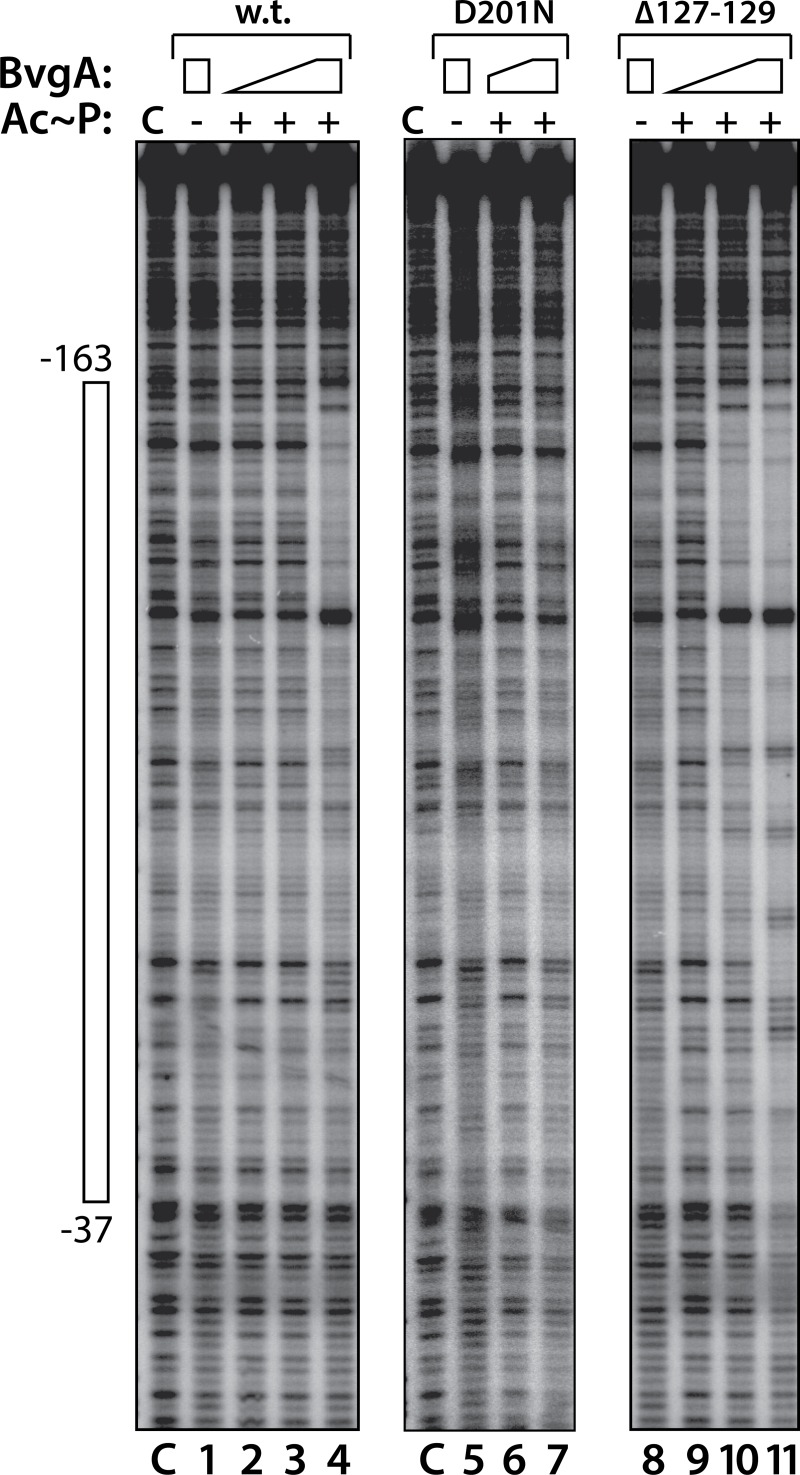
DNase footprinting of BvgA and derivatives on the pertussis toxin promoter. DNase footprints were performed as described in Materials and Methods. BvgA proteins were wild-type (lanes 1–4), BvgA^Δ201N^ (lanes 5–7), and BvgA^Δ127–129^ (lanes 8–11). Lanes designated “C” show the labeled fragment, digested with DNase I, in the absence of any added protein. Lanes 1, 5, and 8 show the digestion pattern obtained when BvgA was added but Ac~P was not. Acetyl phosphate was added to the reactions shown in the remaining lanes, as indicated. Concentrations of BvgA proteins used were 16 nM (lanes 2 and 9), 32 nM (lanes 3, 6, and 10) and 65 nM (lanes 1, 4, 5, 7, 8, and 11). The open bar to the left shows the maximal region protected in lane 11.

Previously, using DNase I footprinting, we visualized direct binding of BvgA, dependent upon its phosphorylation with acetyl phosphate, to P*ptx* [15]. This is shown as well in Fig 3. Protection of P*ptx* DNA was observed only when the highest concentration of wild-type BvgA~P was used. The footprint obtained extended from approximately -163, becoming much weaker closer to the -35 region. This corresponds to a binding affinity that is weaker than that observed at P*fha* by several measures (see discussion). When phosphorylated BvgA^D201N^ was used, as shown in Fig 3, binding to the *ptx* promoter was essentially abolished. This is consistent with the *in vivo* phenotype conferred by the *bvgA*^D201N^ allele, Fha^+^, Ptx^-^. The phosphorylated BvgA^Δ127–129^ protein, on the other hand, showed increased binding to P*ptx*, consistent with the *in vivo* phenotype of higher than wild-type *ptx* expression conferred by the *bvgA*^Δ127–129^ allele in strain BP1322, shown in Fig 1. Protection from DNAse I cleavage comparable to that seen with wild-type BvgA was achieved at a lower concentration and at the highest concentration used, protection was more extensive. At this concentration strong protection extended into the core promoter region and evidence of regularly spaced DNAse I hypersensitive sites was also observed. These observations are consistent with the interpretation that deletion of amino acids 127–129 resulted in a BvgA protein that binds with higher affinity to P*ptx*.

### Six dimers of BvgA~P bind to P*ptx* with a geometry common to BvgA-activated promoters

We previously reported the use of FeBABE-modified BvgA as an affinity cleavage reagent to reveal the precise location and orientation of BvgA monomers and dimers bound to P*fha* [12]. Briefly, we observed head-to-head dimers of BvgA bound at three locations. One corresponded to the high-affinity consensus binding site furthest upstream, with two more downstream, and with the most promoter-proximal site abutting the -35 region. The dimers were spaced every 22 bp on center, demonstrating that they were all bound to the same face of the DNA helix. When we attempted to perform a similar analysis with FeBABE-labeled wild-type BvgA on P*ptx* we were unsuccessful. We attributed this failure to the intrinsic lower affinity of BvgA~P binding to P*ptx*, relative to P*fha*, combined with lower solubility of FeBABE-labeled BvgA, relative to the unlabeled protein. We therefore repeated the FeBABE analysis using the BvgA^Δ127–129^ variant. Clear and interpretable cleavage patterns were obtained using this approach.

**Fig 4:**
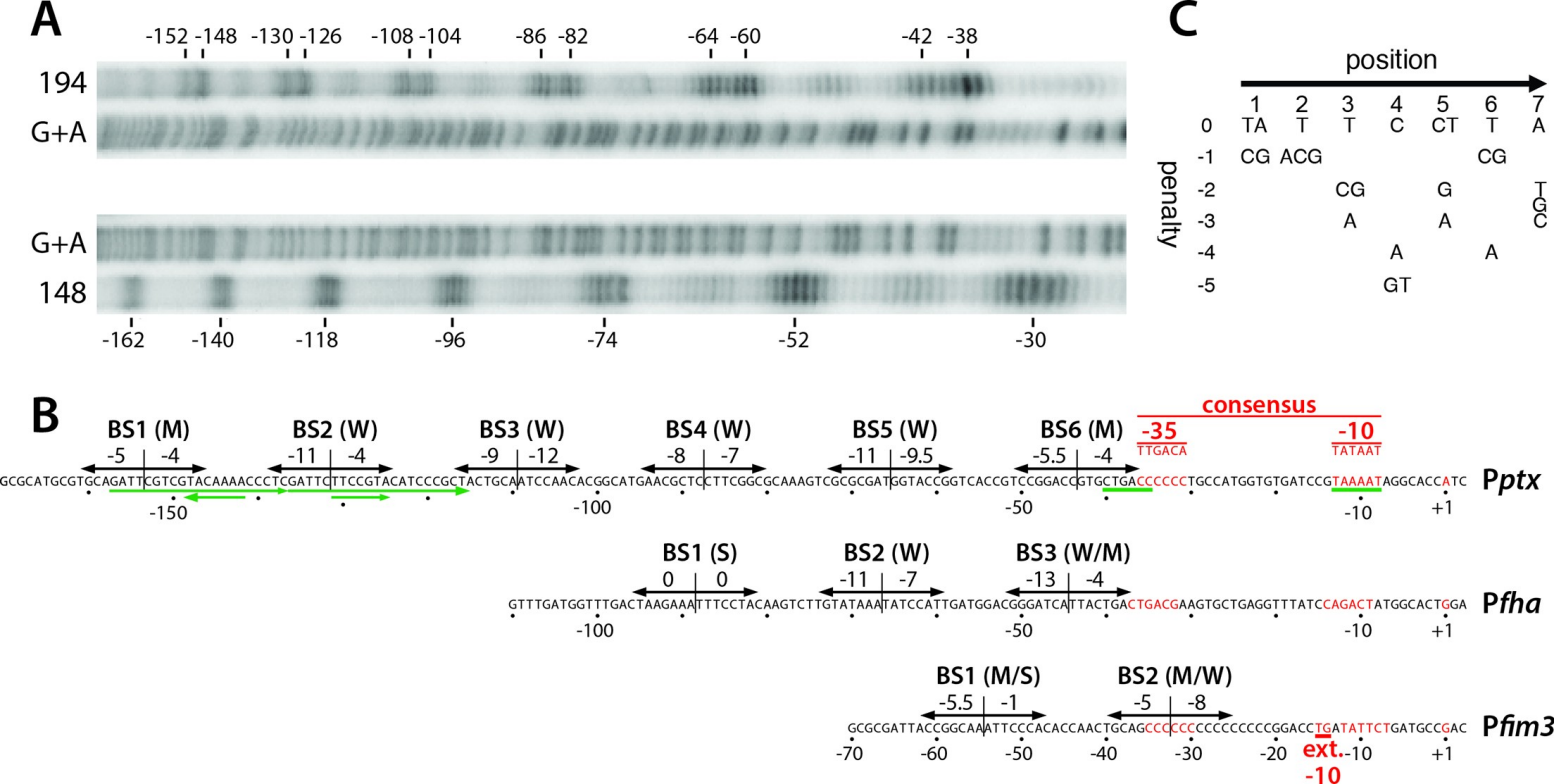
Details of BvgA-binding to the pertussis toxin promoter. **A.** BvgA^Δ127–129^ derivatives in which both naturally occurring cysteine residues had been replaced by alanine and in which either the valine at position 148 or the threonine at position 194 had been replaced with cysteine, were subjected to derivatization with FeBABE and used to reveal the locations of BvgA-binding to the *ptx* promoter using methods previously described for a similar analysis of the *fha* promoter [12]. The protein modified at the 148 position produces cleavages at the outer boundaries of a bound dimer of BvgA~P, while the 194 derivative produces closely spaced cleavages corresponding to the location of the inter-monomer interface. Maxam and Gilbert A + G reactions of the same ^32^P end-labelled P*ptx* DNA fragment were run in parallel for orientation. **B.** DNA sequences of the *ptx* promoter showing the sites of binding of BvgA~P derived from the analysis in panel A. In addition, each heptameric half-site has been scored according to the algorithm presented in panel C, with the scores given above the arrows indicating the binding half-sites. Nucleotides in red indicate core promoter elements, with the consensus sequence shown above. In a similar fashion, P*fha* and P*fim3* are shown for comparison. Green arrows below the P*ptx* sequence indicate two 21 bp imperfect direct repeats and two inverted heptameric imperfect repeats previously cited as potential BvgA binding sites. **C.** Algorithm for predicting binding strength of BvgA-binding half-sites. This algorithm was derived from a study examining the ability of systematically mutated derivatives of the P*fha* primary binding site to bind BvgA~P and to activate transcription [14].

Fig 4A shows the cleavage patterns obtained using BvgA^Δ127–129^ labeled with FeBABE at residue 148 or at residue 194. As previously reported in our analysis of BvgA~P binding to P*fha*, cleavage by the 148 derivative defines the outermost boundaries of dimers of BvgA~P, while that of the 194 derivative produces two cleavages close to the monomer:monomer interface within a dimer [12]. The cleavages we observed with both derivatives at P*ptx* are consistent with this geometry and clearly indicate the presence of six BvgA dimers bound in a similar fashion to P*fha* DNA.

When the cleavage positions were used to determine the DNA sequences corresponding to the specific binding regions for each dimer, the information presented in Fig 4B was obtained. This panel presents the P*ptx* sequence annotated with arrows to indicate specific sequences predicted by FeBABE analysis to be appropriately positioned to interact with each BvgA monomer. Also shown are scores for the predicted relative binding strength of each of these “half-sites”, derived using a previously created algorithm that incorporates the effects of systematic mutagenesis of the high affinity primary binding site of P*fha* [14,19]. As a reference, using this algorithm (Fig 4C), both half-sites of the P*fha* primary binding site have a perfect score of 0, indicating that no mutations were identified in that study that increased binding or transcriptional activation [14]. Analyzing P*ptx* in this way, it can be seen that none of the half-sites identified by the BvgA-FeBABE analysis have a score higher than -4 and no dimer binding site scores (combined half-site scores) are higher than -9. In the schematic diagram presented in Fig 4B, the dimer binding sites, labeled BS1 through BS6, are thus indicated to be of moderate predicted strength (M), as for BS1 and BS6, or weak (W), as for BS2, BS3, BS4, and BS5. Taken together our data support the interpretation that P*ptx* activation is the net result of multiple moderate to low affinity interactions of BvgA~P with DNA, and does not involve high-affinity interactions such as those with the primary binding site of P*fha*.

### Assessment of the individual contributions of the different BvgA-binding sites

**Fig 5:**
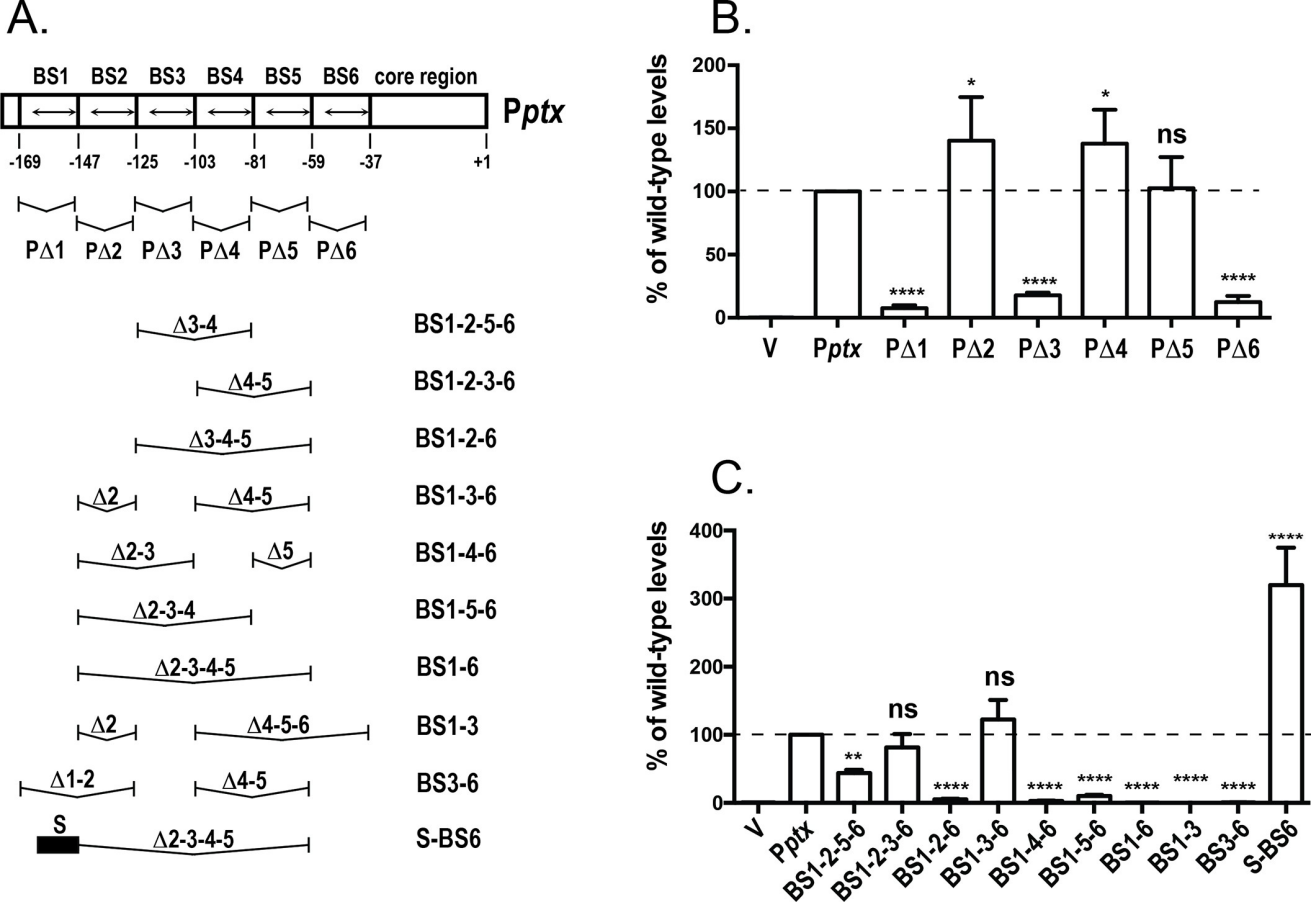
Contribution of P*ptx* BvgA-binding sites to P*ptx* function. **A.** Diagram of BvgA-binding sites within P*ptx* and its derivatives. Boxes delineate the extents of 22-bp deletions removing each of the six binding sites, with the binding sites themselves within each box indicated by a double-headed arrow. Below this schematic are shown the different combinations of these 22 bp segments in different derivatives. The black bar in derivative S-BS6 indicates the presence of the primary binding site from P*fha* (TAAGAAATTTCCTA). **B & C.** Luciferase activity of *B*. *pertussis* strain BP536 carrying ectopically integrated plasmids. Values for the empty pSS3967 control (V) and for promoter-*lux* fusion derivatives harboring the wild type (P*ptx)* and the deletion derivatives shown in panel A are presented. Strains were grown on BG agar at 37°C for 2 days and assayed as described in Materials and Methods. Values were normalized to wild-type P*ptx* and data from at least four assays were used in the calculation of means, standard deviations, as indicated by error bars, and statistical analysis by one-way ANOVA. Outcomes of the latter analysis are presented using the symbols: ns, P > 0.05; *, P ≤ 0.05; **, P ≤ 0.01; ***, P ≤ 0.001; ****, P ≤ 0.0001.

Because the different binding sites were predicted to have different binding affinities, we sought to determine which contributed most to promoter function, and whether some were either essential or dispensable. We approached this question by introducing deletions of one or more binding sites of P*ptx* and assessing the impact those deletions had on P*ptx* activity. To measure P*ptx* activity a 318 bp (-290 to +28 relative to the transcriptional start site) fragment encompassing the complete promoter was cloned into the *lux* fusion vector pSS3967 between the *Eco*RI and *Sal*I sites upstream of the *luxCDABE* operon. Deletion derivatives of this construct were obtained as described in Materials and Methods. The pSS3967 vector is unable to replicate in *B*. *pertussis* and contains a gentamicin resistance gene, the *oriT* site for conjugative transfer of RK2-related plasmids, and a 1.8 kb fragment of the *B*. *pertussis* chromosome. This suicide vector integrates into the *B*. *pertussis* chromosome, via homologous recombination, at a specific location that is unlinked to the promoter under study. In this way, defined promoter fragments can be assayed for their activity, isolated from their natural context, by promotion of *luxCDABE*, resulting in light output. We first deleted each binding region, one at a time, by 22 bp (two helical turns) to ensure that BvgA-binding remained appropriately phased, as depicted in Fig 5A. As presented in Fig 5B, deletion of BS1 (PΔ1) or BS6 (PΔ6) led to a drastic decrease of P*ptx* activity (to 7% in PΔ1 and 12% in PΔ6) while deletion of BS3, as in PΔ3, led to a more moderate decrease (to 18% of wild-type activity). These data indicated that BS1, BS3, and BS6 play crucial roles in P*ptx* function. The deletion of BS2 (PΔ2), BS4 (PΔ4), and BS5 (PΔ5), on the other hand, had no negative effects, in fact leading to somewhat elevated promoter activities of 140%, 137%, and 102% of wild-type, respectively. These observations are reminiscent of a previous deletion study of P*fha*, which contains three binding sites. In that case deletion of either BS1 or BS3 abolished promoter activity, whereas deletion of BS2 alone resulted in a more active promoter [3]. The P*ptx* studies reported here indicate that, similarly, the outermost binding sites, BS1 and BS6, are crucial for activity, and that BS3 may also play an important role, but that BS2, BS4, and BS5 are dispensable.

To further verify the contribution of each binding site to P*ptx* activity, we combined, in various combinations, the single binding site deletions. We first constructed two variants in which the crucial terminal BS1 and BS6 were maintained but in which two internal contiguous binding sites were deleted. The binding sites deleted were BS3 and BS4, as in the P*ptx* variant BS1-2-5-6 or BS4 and BS5, as in the P*ptx* variant BS1-2-3-6. Thus one of these two variants retained BS3 while one did not. As shown in Fig 5C, both retained significant promoter activity. The BS1-2-3-6 derivative displayed almost wild-type levels. BS1-2-5-6 was more severely affected, but still possessed approximately 44% of wild-type activity. These results suggest that BS3, although it contributes to promoter activity, is not essential. Other sites between BS1 and BS6 can serve its function. However BS3 does appear to contribute more than BS2, BS4, or BS5.

We next constructed four P*ptx* variants in which BS1 and BS6 were maintained, but in which only one of the intervening binding sites was maintained. These were named BS1-2-6, BS1-3-6, BS1-4-6, and BS1-5-6. As shown in Fig 5C, in these variants with only three binding sites, only the one with BS3, i.e., BS1-3-6, had significant activity and that one had wild-type levels. These data are consistent with our observations with the single binding site deletions identify BS1, BS3, and BS6 as the most important binding sites, and indicate that BS2, BS4, and BS5 are entirely dispensable. To determine if an active promoter could be constructed with only two binding sites, each of BS1, BS3, and BS6 were individually deleted from the BS1-3-6 variant to create variants BS1-6, BS1-3 and BS3-6. As shown in Fig 5C, none of the resulting variants displayed appreciable activity. Thus, it appears that a functional *ptx* promoter must contain at least three binding sites. This is contrast to P*fha* from which, as described previously [3], BS2 of P*fha* can be deleted to create a promoter with only two binding sites and with no loss of activity. However, it should be noted that, in the case of P*fha*, one of those sites is of higher binding affinity. To examine the effect of an upstream higher affinity site in the context of P*ptx*, we constructed the S-BS6 variant shown in Fig 5A. This variant is similar to the BS1-6 variant, which lacked activity, but BS1 has been substituted with the primary, high affinity, binding site (BS1) of P*fha*. As shown in Fig 5C, this promoter variant, S-BS6, had strong activity, significantly higher than wild-type. Together these data suggest that the need for at least three binding sites in P*ptx* is due, at least in part, to their lower binding affinity. They also indicate that BS6 may play a role in P*ptx* similar to that which BS3 plays in P*fha*.

### BvgAS-regulated activity of P*ptx* depends upon an imperfect -35 region

**Fig 6:**
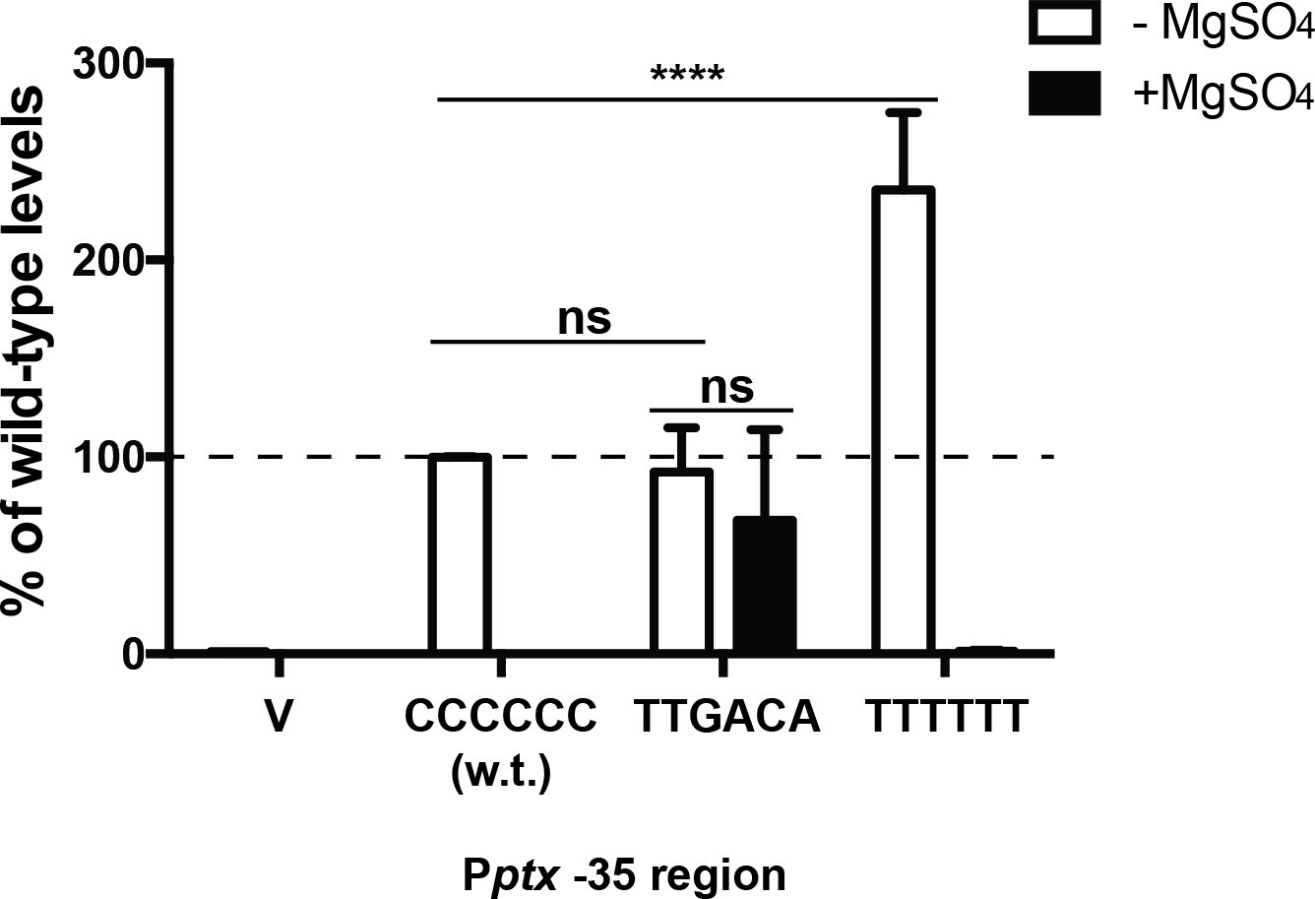
A non-consensus -35 region is required for BvgA-regulated P*ptx* activity. Luciferase activity of *B*. *pertussis* BP536 carrying ectopically integrated plasmids. Values for the empty pSS3967 control (V) and for promoter-*lux* fusion derivatives harboring the wild type (P*ptx*) and -35 substitution derivatives are presented. Strains were grown on BG agar at 37°C for 2 days and assayed as described in Materials and Methods. Values were normalized to wild-type P*ptx* and data from at least four assays were used in the calculation of means, standard deviations, as indicated by error bars, and statistical analysis by one-way ANOVA. Outcomes of the latter analysis are presented using the symbols: ns, P > 0.05; *, P ≤ 0.05; ****, P ≤ 0.0001.

Previous analyses of P*ptx* function by primer extension analysis allowed determination of the transcriptional start site (+1) and thereby suggested an obvious -10 region the appropriate distance from that initiation point [20,21], as shown in Fig 4B. This -10 region (TAAAAT) has a 5/6 match to the consensus TATAAT, with the 3 most crucial bases conserved (underlined) [22]. The location and sequence of a -35 region, on the other hand, has been less apparent. At the time that initial characterizations of P*ptx* were being performed, it was generally held that the -35 element was likely to be the sequence CTGACC, a 4/6 match to the consensus -35 (TTGACA). However, the spacing between this element and the -10 is 21 bp. In recent years, our appreciation of the importance of a more optimal spacing, i.e. close to 17 bp, as a requirement for promoter activity has increased, in part due to a better understanding of its structural basis (see [22] for a review). If optimal 17 bp spacing were to be maintained, the -35 element would have the sequence CCCCCC. While this would at first seem to be an untenable proposal, due to the lack of any real similarity to the consensus sequence for a -35 element, several observations are consistent with this reassignment. 1) The CCCCCC -35 sequence is precisely adjacent to the most downstream binding site, BS6. This configuration is also seen in many of BvgA-activated promoters studied to date, including those driving expression of the *fha*, *bipA*, *bvgR* and *brpL* genes [23]. The previously identified CTGACC, on the other hand would be blocked by the binding of BvgA~P to the B6 binding site. 2) This CCCCCC sequence is found in the -35 regions of three BvgA-activated fimbrial subunit promoters, P*fim2*, P*fim3*, and P*fimX*. At these promoters, due to their unique architecture, the -35 sequence CCCCCC is not adjacent to, but rather is co-centric with, the most downstream BvgA-binding site [7]. Furthermore, at P*fim3*, region 4 of the RNAP sigma subunit was observed, by Fe-BABE labeling and vicinal cleavage, to localize to this segment, in a manner similar to that at a typical -35, -10 promoter [13]. 3) Suboptimal core promoter elements are typical of highly regulated promoters, since perfect consensus elements would lead to a constitutive promoter. In fact, at P*fim3*, changing the -35 sequence CCCCCC to a consensus TTGACA reduced activated promoter activity significantly, and rendered it constitutive at that lower level [7]. To begin to assess the role of this unusual -35 region in P*ptx* function we changed the CCCCCC of P*ptx* to the consensus -35 sequence. As shown in Fig 6, the promoter activity of the derivative containing a consensus -35 element (TTGACA), although comparable to the wild type (CCCCCC) in magnitude, was constitutive, *i*.*e*. unresponsive to MgSO_4_ modulation. When the perfect -35 element (TTGACA) was changed back to a very poor -35 region, although of a different sequence (TTTTTT), higher, regulated, activity was restored. This indicates that it is not the CCCCCC sequence itself that is important, but rather the imperfect nature of its -35 region that is important for P*ptx* BvgA-regulated activity.

### P*ptx* is strongly transcribed in *B*. *pertussis*

**Fig 7:**
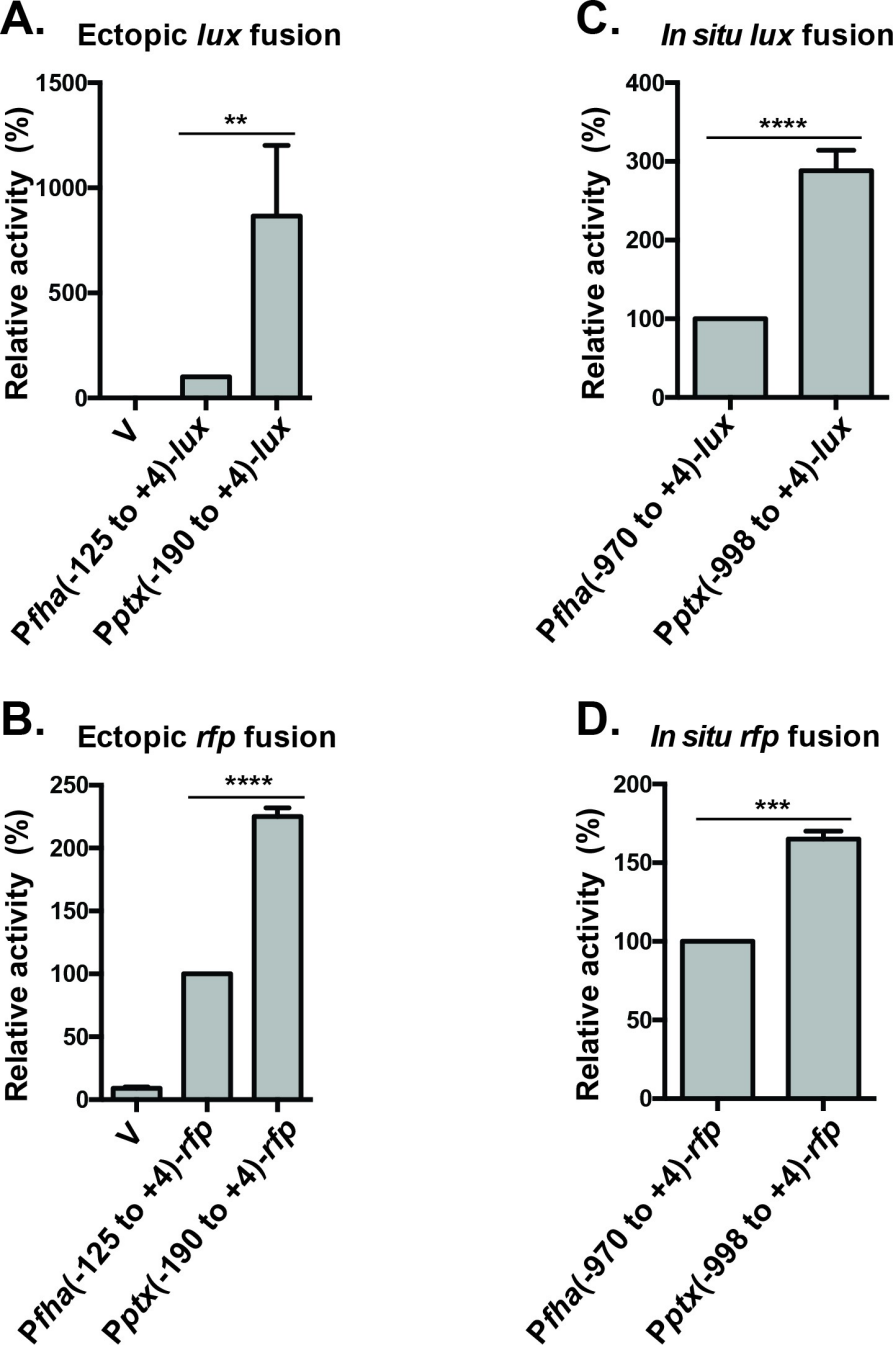
Comparing the relative strengths of P*fha* and P*ptx* by transcriptional fusion. Promoter-*lux* (**A & C**) and promoter-*rfp* (**B & D**) transcriptional fusions were constructed and integrated into the BP536 chromosome. Fusions to the *lux* operon used either pSS3967, to integrate at an ectopic location (**A**), or pSS4162, to integrate *in situ* (**C**). Similarly, transcriptional fusions to *rfp* used either pQC2241 for ectopic insertion (**B**) or pQC2319 for *in situ* insertion (**D**). For the two ectopic constructs “V” indicates insertion of the vector alone. This control is not possible for the *in situ* insertions. The extent of the promoter sequences cloned in each construct are provided as nucleotide coordinates relative to the transcriptional start. *B*. *pertussis* strains carrying these constructs were grown on BG agar at 37°C for 2 days and analyzed for and luciferase and RFP activity as described in Materials and Methods. In each panel activity is reported relative to the P*fha*-promoter fusion and the results of at least four assays were used in the calculation of standard deviations and statistical analysis by an unpaired two-tailed *t* test between two samples. Statistical symbols are: **, P ≤ 0.01; ***, P ≤ 0.001; ****, P ≤ 0.0001.

Previous work showed that *in vitro* transcriptional activities of P*ptx* and P*cya* were lower than that of P*fha*, even at the highest concentrations of BvgA~P used [1]. However, lower activity of P*ptx in vitro* does not necessarily reflect its transcriptional activity *in vivo* in *B*. *pertussis*. To reach maximal transcriptional activity, a higher number of lower affinity binding sites must be occupied. This requires a higher concentration of BvgA~P, one that may not have been achieved *in vitro*, but is experienced *in vivo*. To obtain a measure of the strength of P*ptx in vivo*, we compared its transcriptional activity with that of the strongest known BvgA-regulated promoter, P*fha*. Minimal promoter fragments, extending from a point 30 bp upstream of the upstream boundary of the upstream-most BvgA-binding site to a point 4 bp downstream of the transcriptional start site, were cloned into pSS3967 and introduced into *B*. *pertussis* BP536 as ectopic transcriptional *lux* fusions (-190 to +4 for P*ptx*, and -125 to +4 for P*fha*). This allowed a direct comparison, which indicated that, in this genetic context, luciferase activity directed by P*ptx* is approximately 8-fold higher than that directed by P*fha* (Fig 7A). Recently, we discovered that the *luxCDABE* fusion partner that we have used extensively can, with some promoters, and in a context-dependent manner, lead to levels of luciferase activity that do not accurately represent promoter activity *in vivo* [24,25]. We therefore performed a similar ectopic fusion analysis using *rfp* as an alternative reporter (Fig 7B). We also measured transcriptional activity using these fusions in an *in situ* [25] rather than an ectopic context (Fig 7C & 7D). As shown in Fig 7, in all of these analyses, P*ptx* directed levels of *in vivo* transcription that ranged from 165% to 865% that of P*fha*. Thus, it does appear that BvgA~P concentrations utilized in the assessments of *in vitro* transcription, while sufficient to demonstrate the qualitative nature of BvgA-activation of P*ptx*, may not have allowed a demonstration of the true levels of transcription that this promoter is capable of.

## Discussion

Virulence gene promoters in *Bordetella pertussis* are under the control of the central regulatory operon *bvgAS*. In all cases where it has been examined *in vitro*, BvgA~P has been shown to be both necessary and sufficient for their activation. However, *in vivo*, promoters vary in their responsiveness. Those activated by low BvgA~P concentrations have been termed early genes and those requiring higher levels, late genes. We report here an exploration of structure/function relationships at what is arguably one of the most important late gene promoters, that of the *ptx-ptl* operon. This operon encodes the major virulence factor pertussis toxin and the machinery for its export.

Interaction of P*ptx* with BvgA has been more difficult to characterize than that of the early gene promoter P*fha*, in part due to its lower binding affinity. For example, although BvgA purified from *E*. *coli* was first demonstrated to bind and footprint at P*fha* [26], this protein was not phosphorylated. Binding and footprinting at P*ptx* was not demonstrated until BvgA~P was used [15]. This is because phosphorylation increases the binding affinity of BvgA for its regulated promoters. Similarly, BvgA~P conjugated to FeBABE was used to determine the precise location of BvgA-binding sites in P*fha* [12] and P*fim3* [4,7], but application to P*ptx* has not heretofore been possible, exacerbated by reduced solubility of the Fe-BABE labeled protein. Here we report the isolation of a mutant derivative of BvgA, BvgA^Δ127–129^, with increased affinity for P*ptx*, that overcomes this limitation. Its application has revealed previously unappreciated details of BvgA binding to P*ptx*. Six dimers of BvgA~P bind to this promoter, with the same basic geometry as at P*fha*, *i*.*e*. in a head-to-head configuration, with a distance of 22 bp between dimer centers, and with the furthest downstream binding site abutting the -35 element of the core promoter.

Armed with this information of where, with basepair resolution, BvgA~P binds within P*ptx*, we were able to examine the sequences to which individual molecules bind, in order to assess their predicted binding affinity. We had previously performed an investigation into the sequence requirements for BvgA~P binding by systematically examining the effect of mutations within the primary binding site of P*fha* [14]. This inverted heptad represents an optimal, high-affinity binding site in that no mutations were identified that increased binding or transcriptional activation of P*fha*. All mutations only decreased or had no effect on these two indicators of function. An algorithm was derived to integrate these data and to allow their application to predict binding affinity of other sequences [19]. When we applied this algorithm to the heptad binding half-sites identified by FeBABE analysis of P*ptx*, we found no heptads that scored greater than -4 (Fig 4B). For comparison, at P*fha* a primary binding site composed of two -4-scoring half-sites corresponded to just detectable binding and transcriptional activation [14]. In this paper we have referred to dimer binding sites, each composed of two inverted half sites, with a total score of -9 or -10 as medium-affinity sites (M) and those with lower scores as weak, low-affinity, sites (W) (Fig 4B).

In addition to illuminating an experimental path forward, our findings correct a misinterpretation about BvgA binding to P*ptx* that has existed for years. Previously, based on the upstream extent of sequences bound by BvgA~P, deletion analyses and, admittedly subjective, sequence-gazing for heptad sequences that matched the P*fha* primary binding site sequences, two heptads were identified that were in an inverted orientation to each other, separated by 10 bp, and embedded within to direct repeats 21 bp in length. These features were cited by many, ourselves included [15], as potentially important BvgA binding sites in P*ptx* (see Fig 4B). Our current analysis, combining a precise determination of where each molecule of BvgA~P binds, together with an objective determination of the functional fit of the sequences bound, gives a more accurate picture of the factors dictating BvgA binding to P*ptx*.

According to the P*ptx* architecture illustrated in this work (Fig 4B), the furthermost upstream (BS1) and downstream (BS6) dimer binding sites have the highest, although moderate, binding affinity, while those in between (BS2 –BS5) are of lower affinity. Our deletion analysis indicates that the two moderate-affinity sites are essential. As regards the most upstream site, BS1, a reasonable interpretation of its essentiality is that it plays a role similar to that of the primary binding site of P*fha*, *i*.*e*. as a nucleation point for further cooperative binding of BvgA dimers extending downstream, eventually to the -35 core promoter region. The most promoter proximal BvgA binding site, BS6, on the other hand, presumably hosts the BvgA dimer interacting directly with the RNA polymerase sigma subunit and thus may also require some degree of specificity. It is not known if this site, BS6 in P*ptx*, has enabling features other than affinity of the appropriate magnitude. We speculate that in this position, a binding affinity that is too high is detrimental to promoter activity. We envisage that, a BvgA~P dimer bound to this site requires some structural flexibility in order to facilitate productive interaction with RNAP or to allow promoter clearance and that such flexibility could be reduced by high affinity DNA-binding. For binding sites BS2-5, the four low affinity binding sites between BS1 and BS6, any one will suffice, in a qualitative sense, to allow promoter activity, if the other three are deleted. However, BS3 is most capable in this regard since BS1-3-6 has full activity while BS1-2-6, BS1-4-6, and BS1-5-6 have only partial activity. The features of BS3 that are the basis of this differential ability are unknown at this time.

In keeping with the apparent requirement for promoter proximal binding of BvgA that is not overly strong, we also show here that an optimal -35 element, that presumably dictates higher affinity binding of RNAP, is also less productive of regulated promoter activity. The P*ptx* sequence that occupies the -35 region location is CCCCCC and presumably dictates weak, if any, inherent binding of region 4 of the primary sigma factor, known to interact with the -35 element via a HTH motif. Changing the CCCCCC to the consensus -35 sequence TTGACA rendered P*ptx* largely unregulated. Changing it back to TTTTTT restored higher, regulated P*ptx* activity. This suggests that, as we propose for the promoter proximal BvgA dimer, overly tight binding of RNAP is counterproductive for overall promoter activity, as well as its dependence on BvgA~P. These results are also supportive of our reinterpretation of both the location and sequence of the -35 region of P*ptx*.

The BvgA^Δ127–129^ protein conjugated to Fe-BABE represents a useful new tool to investigate promoter architecture of BvgA-regulated promoters that lack strong binding sites. Its application, as reported here, has led us to a greatly improved understanding of structure/function relationships at P*ptx* and will do so at other low affinity promoters. Indeed, initial application of this tool to the late promoter P*cya* has revealed a unique binding configuration in that the most promoter proximal of the 4 dimers bound deviates from the 22 bp spacing seen at all other promoters [23]. On the other hand, it is natural to wonder about why the Δ127–129 mutation negatively affects P*fha* function when it has the opposite effect at the low affinity promoter P*ptx*. Several possibilities immediately present themselves. It may be, in fact, that the intrinsic higher affinity of P*fha*, in combination with a BvgA protein of increased affinity, result in less productive interactions precisely due to their higher affinity for each other. In one scenario BvgA~P, stabilized by DNA-binding, oligomerizes past the binding site of the third dimer, BS3, and obstructs access of RNA polymerase to the -35 element. In another, BvgA^Δ127–129^ bound to BS3 interacts so tightly that interaction with RNA polymerase is less productive, either because this BvgA molecule cannot adopt a slightly less constrained conformation, thus allowing RNAP binding, or because promoter clearance is inhibited.

The precise structural bases of the higher DNA-binding affinity of the BvgA^Δ127–129^ protein remain to be elucidated. However, it has proven useful in the past to model the BvgA primary sequence on known crystal structures of NarL, a homologue of BvgA [12,13]. A structure of the complete unphosphorylated NarL protein suggests that DNA binding activity of the C-terminal domain is sterically hindered by the N-terminal response-regulator domain [27]. A model for NarL activation, supported by NMR spectroscopy, proposes that conformational changes in the N-terminal domain, resulting from phosphorylation, may alleviate this hindrance, allowing the C-terminal domain to then bind and activate regulated promoters [28], The linker between the two domains is bounded on the N-terminal side by the sixth alpha helix of the N-terminal receiver domain and it is within this predicted helix in BvgA that three amino acids are deleted in the BvgA^Δ127–129^ protein. This would be predicted to result in a net rotation of the linker and C-terminal domain of 60 degrees along the axis of the alpha-6 helix. While it is not possible to precisely predict the resulting structure, we hypothesize that this results in a more open conformation of BvgA^Δ127–129^. Although this mutation is not sufficient to render BvgA constitutively active, i.e. independent of the need for phosphorylation, it may serve to lower energy barriers along the path to adopting a DNA-binding-competent conformation. Alternatively, this more open conformation could promote dimer-dimer interactions, facilitating BvgA dimerization along the DNA, to then allow productive interactions with RNAP. These two possibilities are not mutually exclusive and do not represent the only conceivable hypotheses.

A quantitative determination of P*ptx* activity *in vivo* has been challenging. We used gene fusions to both *luxCDABE* and *rfp* in either ectopic (isolated) or *in situ* (native) genetic contexts to compare intrinsic promoter strength of P*ptx* to that of P*fha*, one of the strongest known Bvg-activated promoters. By all of these determinations, P*ptx* is more active than P*fha* (Fig 7). Thus, in spite of an architecture that incorporates superficially suboptimal aspects, the *ptx* promoter achieves high levels of transcription of the structural genes for pertussis toxin and its secretory apparatus, at levels of BvgA~P encountered in the Bvg^+^ mode.

Our findings have provided a more detailed picture of how the promoter architecture of P*ptx* dictates its relatively low responsiveness to BvgA~P concentration. A combination of lower affinity sites, the best corresponding to just detectable expression in the context of P*fha*, are apparently only filled when BvgA~P levels are higher than those required to induce “early” promoters such as P*fha*. In laboratory experiments such conditions are achieved only hours after induction of the Bvg^+^ mode by temperature shift, or, in unvarying growth conditions, at lower concentrations of negative modulators such as MgSO_4_ or nicotinic acid. Regarding these differences in behavior between P*fha* and P*ptx*, it is tempting to ask “Why?”. It has been postulated that these differences dictate a program of gene expression following transmission to the respiratory tract of a new host whereby the products of early genes, primarily adhesins, are produced first, allowing incoming bacteria to attach and establish a beachhead. Then at later times, toxins are induced that can counteract host immune responses [8]. While this is a plausible and attractive scenario, the evolutionary context of the *ptx* promoter should also be considered. The acute human pathogen *B*. *pertussis* has apparently evolved relatively recently from a latest common ancestor more closely related to the chronic veterinary pathogen *B*. *bronchiseptica* [29]. One of the key attributes acquired during this evolution is the ability to express pertussis toxin in the mammalian host. Many *B*. *bronchiseptica* strains harbor intact *ptx* and *ptl* operons. However they have not reliably been observed to produce pertussis toxin. The key difference between these two species in this regard resides in their promoter regions. Although they are generally similar in DNA sequence, 18 SNPs are present that dictate this important phenotypic divergence. That the structural genes for pertussis toxin and its secretion apparatus remain functional in *B*. *bronchiseptica* strains that contain them is evidenced by the fact that replacement of the *B*. *bronchiseptica ptx* promoter with one from *B*. *pertussis* resulted in regulated production and secretion of active pertussis toxin [30]. Precisely which SNPs are responsible for this key development in evolution remains to be reported. However this evolutionary picture is not really consistent with a view of the pertussis toxin promoter as a highly adapted regulatory apparatus contributing to an optimized temporal pattern of gene expression. It is more like the panda’s (sixth) thumb, an example of the principle that novel structures in evolution arise by natural selection, out of need, and from existing structures, rather than according to an elegant program of development. In the words of Stephen J. Gould “Like the parts of Darwin’s orchids they are familiar bits of anatomy remodeled for a new function.” In this case the “new function” is the production of pertussis toxin in the mammalian host and the “familiar bits of anatomy” are represented by the *B*. *bronchiseptica ptx* promoter.

## Materials and methods

### Bacterial strains and culture conditions

Bacterial strains and plasmids used in this study are listed in Table 1. *E*. *coli* strains were grown in LB broth or on LB agar. Antibiotic concentrations used for *E*. *coli* strains were 100 μg/ml ampicillin and 5 μg/ml gentamicin. *B*. *pertussis* strains were grown on BG agar [31]. Antibiotic concentrations used for *B*. *pertussis* strains were 50 μg/ml streptomycin, 25 μg/ml kanamycin, and 10 μg/ml gentamicin.

**Table 1:** Strains and plasmids used in this work.

Strain or plasmid	Relevant features	Source or reference
***E*. *coli***		
DH5α	High-efficiency transformation	Bethesda Research Laboratories
SM10	Conjugation proficient *E*. *coli* donor strain	[39]
***B*. *pertussis***		
Tohama I	Patient isolate	[40]
BP536	Tohama I, Str^R^, Nal^R^	[31]
BP953	BP536, *fhaB-lacZ*, *ptx-phoA*	[17]
BP1056	Pho^-^, Lac^+^, Hly^-^ mutant of BP953 (*bvgA*^D201N^)	[17]
BP1286	Pho^+^ mutant of BP1056 (*bvgA*^D201N, Δ127–129^)	This study
BP1318	BP953 *bvgA*^D201N^, Lac^+^, Pho^-^, created by allelic exchange (pSS2423). Clean version of BP1056.	This study
BP1324	BP953 *bvgA*^D201N, Δ127–129^, Lac^+^, Pho^+^, created by allelic exchange (pSS2429). Clean version of BP1286.	This study
BP1322	BP953 *bvgA*^Δ127–129^, Lac^+^, Pho^+^, created by allelic exchange (pSS2427)	This study
QC3216	(BP536 Δ*bvgA*::P2*bvgS*)	[9]
**Plasmids**		
pSS1827	Helper plasmid for triparental mating, RP4 *tra* genes, Amp^R^	[41]
pTTQ8	P*tac* expression vector	[34]
pSS1894	Suicide vector, *oriT*, *gen*, *amp*,	[33]
pSS2000	pSS1894::*rpsL*	This study
pSS1574	pTTQ8::*phoA*	This study
pSS2072	pSS1574::*aph*	This study
pSS2075	Suicide vector, *oriT*, *gen*, *amp*, ‘*phoA*-*aph*	This study
pSS2197	*bvgA* allelic retrieval vector	[18]
pSS2401	Derived by allelic retrieval of *bvgA* from BP1286 using pSS2197	This study
pSS2423	pSS2401 derivative containing *bvgA*^D201N^	This study
pSS2427	pSS2401 derivative containing *bvgA*^Δ127–129^	This study
pSS2429	pSS2401 derivative containing *bvgA*^D201N, Δ127–129^	This study
pSS3967	Ectopic *luxCDABE* promoter assay vector, Amp^r^ Gen^r^	[7,25]
pQC1114	pSS3967::WT P*ptx* (-290 to +28 relative to P*ptx* +1)	This work
pQC1384	pSS3967::PΔ1 (-290 to +28 relative to P*ptx* +1)	This work
pQC1385	pSS3967::PΔ2 (-290 to +28 relative to P*ptx* +1)	This work
pQC1115	pSS3967::PΔ3 (-290 to +28 relative to P*ptx* +1)	This work
pQC1386	pSS3967::PΔ4 (-290 to +28 relative to P*ptx* +1)	This work
pQC1119	pSS3967::PΔ5 (-290 to +28 relative to P*ptx* +1)	This work
pQC1383	pSS3967::PΔ6 (-290 to +28 relative to P*ptx* +1)	This work
pQC1116	pSS3967::BS1-2-5-6 (-290 to +28 relative to P*ptx* +1)	This work
pQC1120	pSS3967::BS1-2-3-6 (-290 to +28 relative to P*ptx* +1)	This work
pQC1117	pSS3967::BS1-2-6 (-290 to +28 relative to P*ptx* +1)	This work
pQC1468	pSS3967::BS1-3-6 (-290 to +28 relative to P*ptx* +1)	This work
pQC1584	pSS3967::BS1-4-6 (-290 to +28 relative to P*ptx* +1)	This work
pQC1585	pSS3967::BS1-5-6 (-290 to +28 relative to P*ptx* +1)	This work
pQC1469	pSS3967::BS1-6 (-290 to +28 relative to P*pt*x +1)	This work
pQC1488	pSS3967::BS1-3 (-290 to +28 relative to P*ptx* +1)	This work
pQC1489	pSS3967::BS3-6 (-290 to +28 relative to P*ptx* +1)	This work
pQC1490	pSS3967::S-BS6 (-290 to +28 relative to P*ptx* +1)	This work
pQC1467	pSS3967::TTGACA (-290 to +28 relative to P*ptx* +1)	This work
pQC1487	pSS3967::TTTTTT (-290 to +28 relative to P*ptx* +1)	This work
pQC1557	pSS3967:: WT P*fha*- (BS+30, -124 to +4)-*lux* in pSS3967	[9]
pQC1552	pSS3967::WT P*ptx*-BS+30 (-190 to +4 relative to P*ptx* +1)	This work
pQC2241	Ectopic *rfp* promoter assay vector, Ampr Genr	This work
pQC2306	pQC2241::WT P*fha*-BS+30 (-125 to +4 relative to P*fha* +1)	This work
pQC2307	pQC2241:: WT P*ptx*-BS+30 (-190 to +4 relative to P*ptx* +1)	This work
pSS4162	*In situ luxCDABE* promoter assay vector, Amp^r^ Gen^r^	[25]
pQC2623	pSS4162:: WT P*fha* (-970 to +4 relative to P*fha* +1)	This work
pQC2624	pSS4162::WT P*ptx* (-998 to +4 relative to P*ptx* +1)	This work
pQC2319	*In situ rfp* promoter assay vector, Ampr Genr	[25]
pQC2626	pQC2319:: WT P*fha* (-970 to +4 relative to P*fha* +1)	This work
pQC2627	pQC2319:: WT P*ptx* (-998 to +4 relative to P*ptx* +1)	This work
pQC1883	*B*. *pertussis* expression vector, Kan^r^	[9]
pSS4983	pQC1883::*bvgA*, Kan^r^	[9]
pQC1894	pQC1883::*bvgA*^Δ127–129^	This work

### Construction of plasmids

*B*. *pertussis* BP536 (Tohama I lineage) genomic DNA was used as the template for PCR amplification unless otherwise specified. The sequences of oligonucleotides and synthetic DNA fragments used for plasmid construction are provided in S1 Table. The DNA sequences of P*ptx*, P*fha* and their derivatives generated in the study are provided in Supplemental S1 Fig.

To measure transcriptional activity of promoters of interest, in single copy, in an ectopic but constant chromosomal location in *B*. *pertussis*, the *luxCDABE* promoter assay vector pSS3967 was used [7,25]. For example plasmid pQC1114 contains a PCR-generated wild-type P*ptx* fragment (-290 to +28 relative to P*ptx* +1) cloned between the *Eco*RI and *Sal*I sites upstream of *luxCDABE* in pSS3967. Plasmid pQC1552 is similar to pQC1114 but the P*ptx*-containing fragment is smaller, comprising sequences from -190 to +4, relative to P*ptx* +1. P*ptx* promoter variants derived from these two plasmids were created using a site-directed mutagenesis procedure that takes advantage of the characteristics of the type IIS restriction enzyme *Bsa*I, as described by Stemmer and Morris except that inverse PCR was not performed [32].

To allow the assessment of transcriptional activities using red fluorescence protein as a reporter we constructed the ectopic *rfp* promoter assay vector pQC2241 as follows. A 153 bp synthetic DNA fragment (gBlock, Integrated DNA Technologies, Inc.) (shown in S1 Table) containing a cleavage site for *Mfe*I at one end and *Eco*RI and *Sal*I sites at the other end was digested with *Mfe*I and *Sal*I and ligated into pSS3967 that had been digested with *Eco*RI and *Sal*I to yield an intermediate plasmid. This plasmid was digested with *Eco*RI and *Blp*I to remove *luxCDABE* and the remaining plasmid backbone was ligated with *Eco*RI and *Blp*I digested *rfp* gBlock (S1 Table) to yield plasmid pQC2241. Two derivatives of pQC2241 were created by cloning PCR-generated *Eco*RI-*Sal*I fragments between its *Eco*RI and *Sal*I sites. Plasmid pQC2307 contains the wild-type P*ptx* from -190 to +4, relative to P*ptx* +1 and plasmid pQC2306 contains the wild-type P*fha* from -125 to +4, relative to P*fha* +1.

To allow the measurement of the transcriptional activity of promoters in an *in situ* rather than an ectopic location, deletion derivatives of the *luxCDABE* reporter plasmid pSS3967 and the *rfp* reporter plasmid pQC2241 were used. These vectors, pSS4162 and pQC2319, respectively, no longer contain a fragment of *B*. *pertussis* genomic DNA [25]. Instead insertion by homologous recombination is mediated by DNA sequences upstream of the promoters of interest. This leads, after insertion, to juxtaposition of the reporter gene downstream of the promoter of interest, with all upstream sequences intact. To this end, a P*ptx* fragment (-998 to +4, relative to P*ptx* +1) was cloned into pSS4162 and pQC2319 to create pQC2624 and pQC2627, respectively. In a similar fashion, a P*fha* fragment (-970 to +4, relative to P*fha* +1) was cloned into pSS4162 and pQC2319 to create pQC2623 and pQC2626, respectively.

To allow overexpression of BvgA^Δ127–129^ in *B*. *pertussis*, the IPTG-inducible *lac* promoter-based expression vector pQC1883 was used [9]. A *Sal*I-*Bam*HI PCR fragment containing the *bvgA*^Δ127–129^ gene was generated using primers Q1841 & 3100 (S1 Table), and DNA template from *B*. *pertussis* strain BP1322 and cloned between the *Sal*I and *Bam*HI sites downstream of P*lac* in plasmid pQC1883. The resulting plasmid pQC1894 was then introduced by conjugation into the *B*. *pertussis* strain QC3216 (BP536::*ΔbvgA*-P2*bvgS*) as described previously [9]. The previously described plasmid pSS4983 containing the wild-type *bvgA* gene [9] was also introduced into QC3216 for use as a control expressing wild-type BvgA.

The suicide vector construct pSS2075, whose use is described below, is a derivative of pSS1894, a pBR322-based plasmid, marked with gentamicin and ampicillin resistance genes, and containing *oriT* of the broad host range conjugative plasmid RK2, whose construction has been described previously [33]. The derivative pSS2000 was created by cloning a 700 bp *Sal*I fragment, containing the *E*. *coli rpsL* gene, together with its native promoter, into the *Xho*I site of pSS1894. A PCR fragment of the *E*. *coli phoA* gene was cloned into the vector pTTQ8 [34], between the *Xma*I and *Hin*dIII sites, to create pSS1574. A promoter-less kanamycin resistance gene was cloned as a PCR fragment downstream of the *phoA* gene of pSS1574 to create pSS2072. In a final step, a fragment derived from pSS2072, containing the *phoA* gene truncated at its 5’ end as well as the downstream kanamycin resistance gene, was cloned into pSS2000 to create pSS2075. This plasmid is capable of conjugative transfer from *E*. *coli* donors into *B*. *pertussis* strains, where it is unable to replicate. Imposition of selection for gentamicin resistance will select for exconjugants in which the plasmid has integrated, via homologous recombination, into a *phoA* gene, if present, thus placing the kanamycin resistance gene under the same transcriptional signals as the *phoA* gene.

### Isolation of the Δ127–129 allele of *bvgA*

Strains harboring the Δ127–129 mutant allele of *bvgA* were obtained as follows. The *B*. *pertussis* strain BP1056 is a mutant derivative of the double transcriptional fusion strain BP953 (BP536 *fha-lacZ*, *ptx-phoA*). BP1056 was obtained after nitrosoguanidine mutagenesis of BP953 and screening for Lac^+^ Pho^-^ colonies (Fha^+^, Ptx^-^). It harbors the *bvgA*^D201N^ mutation and its isolation has been described elsewhere [17]. The *ptx-phoA* fusion in BP1056 was converted to a kanamycin-resistance gene transcriptional fusion by conjugative transfer of the suicide vector construct pSS2075 and selection for gentamicin resistance encoded on the vector backbone. This plasmid contains a partial *phoA* gene truncated at the 5’ end and placed upstream of a promoterless *aph* gene. Insertion of this plasmid by recombination via its region of homology in the *phoA* gene results in the placement of the *aph* gene, specifying kanamycin resistance, downstream of *ptx-phoA* thus making the kanamycin resistant phenotype dependent on transcription from P*ptx*. Spontaneous mutants of BP1056::pSS2075 that regained expression of the *ptx-phoA* fusion were selected on BG agar containing kanamycin and screened for alkaline phosphatase activity by perfusion of colony lifts with XP (5-bromo-4-chloro-3-indolyl-phosphate), as previously described [17]. The pSS2075 insertion in these strains was subsequently lost by homologous recombination and plasmid segregation. One mutant strain so derived was named BP1286. BP1286 was subjected to allelic retrieval of the *bvgA* gene using pSS2197 as previously described [18], resulting in the isolation of pSS2401. Briefly, this procedure is similar to allelic exchange in its first step in that a suicide plasmid is introduced into a strain of interest and integrants, arising by homologous recombination between cloned and chromosomal sequences, are isolated. In the allelic retrieval approach the plasmid contains sequences flanking the area to be recovered, which is itself deleted and replaced with *sacB*. After a second recombination event the plasmid is liberated. If both recombination events occurred on the same side of the *sacB* marker, the wild-type is maintained in the chromosome and the deletion on the plasmid. However, if the crossovers occur on different sides the chromosomal allele will now contain the *sacB*-marked deletion and the plasmid will contain the intact chromosomal sequences between the flanking regions of homology. The liberated non-replicating plasmids are captured in *E*. *coli* by transformation or conjugation and selection for sucrose resistance ensures that only successful allelic retrieval events are captured. In this way plasmid pSS2401 was isolated, resulting in a plasmid containing the *bvgA* gene from BP1286, in its native context relative to wild-type *fhaB’* and *bvgS’* flanking sequences, in the allelic exchange vector pSS1129 [35]. DNA sequence analysis indicated that the *bvgA* gene in pSS2401, retrieved from BP1286, contained a mutation in addition to the D210N mutation present in the parental BP1056. This mutation was a deletion of three codons encoding amino acid residues 127–129. Fragment swapping cloning was performed with pSS2401 and similar plasmids that lacked either the D201N or Δ127–129 mutations. In this way, plasmids pSS2423, pSS2427, and pSS2429, were constructed, which contained D201N, Δ127–129, or both mutations, respectively. These plasmids were used to reintroduce each *bvgA* allele into a clean BP953 genetic background by allelic exchange, as described [35]. In this way, BP1318, BP1322, and BP1324 were created.

### *In vivo* luciferase and RFP activity assays

*B*. *pertussis* strains harboring promoter-*lux* fusions or promoter-*rfp* fusions were analyzed for luciferase and RFP activities *in vivo* as previously described [25]. Data, averaged from at least 4 assays, were presented as arbitrary relative luminescence units (RLU; photons per second) or fluorescence units (RFU), or are presented relative to the wild-type promoter control strain or other luminescent or fluorescent strains used as a reference on a given plate. One-way analysis of variance (ANOVA) and unpaired two-tailed t test were carried out using Prism 6 software.

### Phos-tag gel electrophoresis analysis of BvgA phosphorylation

*B*. *pertussis* strains harboring BvgA-expressing plasmids were cultured in PLB liquid media [36] and induced with 1mM IPTG. Collected cells were analyzed for BvgA phosphorylation using Phos-tag gel electrophoresis followed by anti-BvgA Western blot analyses as described previously [9] and ImageJ software for the quantification of detected BvgA.

### Alkaline phosphatase and beta-galactosidase assays of *B*. *pertussis* strains

Beta-galactosidase assays and alkaline phosphatase assays of *B*. *pertussis* strains were performed as previously described [33] using variations of the methods of Miller [37] and Brickman et al. [38], respectively.

### DNase I and FeBABE footprinting

DNase I footprinting was performed as previously described [2]. FeBABE analysis of BvgA~P binding to the *ptx* promoter was performed as previously described [12].

## Supporting information

S1 Fig DNA sequences of P*ptx*, P*fha*, and their derivatives used in this study. **A.** Wild-type P*ptx*. Binding sites for BvgA~P are underlined. Nucleotides in blue denote core promoter elements +1, -10 and -35. **B & C.** Deletion derivatives of P*ptx* with one (B) or more (C) BvgA binding sites deleted, as denoted by hyphens. In the variant S-BS6, yellow highlighting indicates the primary binding site from P*fha*. **D.** P*ptx* variants with altered -35 regions, shown in red. **E.** Fragments used for comparisons of the strength of P*ptx* and P*fha*. The P*fha* strong binding site is highlighted.
(TIF)

S1 Table Oligos and DNA fragments used for cloning. (XLSX)

## References

[1] Steffen P, Goyard S, Ullmann A. Phosphorylated BvgA is sufficient for transcriptional activation of virulence-regulated genes in Bordetella pertussis. EMBO J. 1996;15(1):102–9. 8598192 PMC449922

[2] Boucher PE, Murakami K, Ishihama A, Stibitz S. Nature of DNA binding and RNA polymerase interaction of the Bordetella pertussis BvgA transcriptional activator at the fha promoter. J Bacteriol. 1997;179(5):1755–63. 10.1128/jb.179.5.1755-1763.1997 9045838 PMC178891

[3] Boucher PE, Yang MS, Schmidt DM, Stibitz S. Genetic and biochemical analyses of BvgA interaction with the secondary binding region of the fha promoter of Bordetella pertussis. J Bacteriol. 2001;183(2):536–44. 10.1128/JB.183.2.536-544.2001 11133947 PMC94909

[4] Boulanger A, Moon K, Decker KB, Chen Q, Knipling L, Stibitz S, et al Bordetella pertussis fim3 gene regulation by BvgA: phosphorylation controls the formation of inactive vs. active transcription complexes. Proc Natl Acad Sci U S A. 2015;112(6):E526–35. 10.1073/pnas.1421045112 25624471 PMC4330726

[5] Kinnear SM, Boucher PE, Stibitz S, Carbonetti NH. Analysis of BvgA activation of the pertactin gene promoter in Bordetella pertussis. J Bacteriol. 1999;181(17):5234–41. 10464192 10.1128/jb.181.17.5234-5241.1999 PMC94027

[6] Williams CL, Boucher PE, Stibitz S, Cotter PA. BvgA functions as both an activator and a repressor to control Bvg phase expression of bipA in Bordetella pertussis. Mol Microbiol. 2005;56(1):175–88. 10.1111/j.1365-2958.2004.04526.x 15773988

[7] Chen Q, Decker KB, Boucher PE, Hinton D, Stibitz S. Novel architectural features of Bordetella pertussis fimbrial subunit promoters and their activation by the global virulence regulator BvgA. Mol Microbiol. 2010;77(5):1326–40. 10.1111/j.1365-2958.2010.07293.x 20662776 PMC2975811

[8] Scarlato V, Arico B, Prugnola A, Rappuoli R. Sequential activation and environmental regulation of virulence genes in Bordetella pertussis. EMBO J. 1991;10(12):3971–5. 1718746 10.1002/j.1460-2075.1991.tb04967.x PMC453138

[9] Boulanger A, Chen Q, Hinton DM, Stibitz S. In vivo phosphorylation dynamics of the Bordetella pertussis virulence-controlling response regulator BvgA. Mol Microbiol. 2013;88(1):156–72. 10.1111/mmi.12177 23489959 PMC3608721

[10] Stibitz S. Mutations affecting the alpha subunit of Bordetella pertussis RNA polymerase suppress growth inhibition conferred by short C-terminal deletions of the response regulator BvgA. J Bacteriol. 1998;180(9):2484–92. 9573202 10.1128/jb.180.9.2484-2492.1998 PMC107192

[11] Veal-Carr WL, Stibitz S. Demonstration of differential virulence gene promoter activation in vivo in Bordetella pertussis using RIVET. Mol Microbiol. 2005;55(3):788–98. 10.1111/j.1365-2958.2004.04418.x 15661004

[12] Boucher PE, Maris AE, Yang MS, Stibitz S. The response regulator BvgA and RNA polymerase alpha subunit C-terminal domain bind simultaneously to different faces of the same segment of promoter DNA. Mol Cell. 2003;11(1):163–73. 10.1016/s1097-2765(03)00007-8 12535530

[13] Decker KB, Chen Q, Hsieh ML, Boucher P, Stibitz S, Hinton DM. Different requirements for sigma Region 4 in BvgA activation of the Bordetella pertussis promoters P(fim3) and P(fhaB). J Mol Biol. 2011;409(5):692–709. 10.1016/j.jmb.2011.04.017 21536048 PMC3141349

[14] Boucher PE, Yang MS, Stibitz S. Mutational analysis of the high-affinity BvgA binding site in the fha promoter of Bordetella pertussis. Mol Microbiol. 2001;40(4):991–9. 10.1046/j.1365-2958.2001.02442.x 11401705

[15] Boucher PE, Stibitz S. Synergistic binding of RNA polymerase and BvgA phosphate to the pertussis toxin promoter of Bordetella pertussis. J Bacteriol. 1995;177(22):6486–91. 10.1128/jb.177.22.6486-6491.1995 7592424 PMC177499

[16] Marques RR, Carbonetti NH. Genetic analysis of pertussis toxin promoter activation in Bordetella pertussis. Mol Microbiol. 1997;24(6):1215–24. 10.1046/j.1365-2958.1997.4371792.x 9218770

[17] Stibitz S. Mutations in the bvgA gene of Bordetella pertussis that differentially affect regulation of virulence determinants. J Bacteriol. 1994;176(18):5615–21. 10.1128/jb.176.18.5615-5621.1994 8083156 PMC196763

[18] Stibitz S. Allelic retrieval: a scheme to facilitate the repeated isolation of a specific segment of the Bordetella pertussis chromosome. Gene. 1998;208(2):183–9. 10.1016/s0378-1119(97)00643-4 9524261

[19] Merkel TJ, Boucher PE, Stibitz S, Grippe VK. Analysis of bvgR expression in Bordetella pertussis. J Bacteriol. 2003;185(23):6902–12. 10.1128/JB.185.23.6902-6912.2003 14617654 PMC262712

[20] Locht C, Keith JM. Pertussis toxin gene: nucleotide sequence and genetic organization. Science. 1986;232(4755):1258–64. 10.1126/science.3704651 3704651

[21] Gross R, Rappuoli R. Pertussis toxin promoter sequences involved in modulation. J Bacteriol. 1989;171(7):4026–30. 10.1128/jb.171.7.4026-4030.1989 2544567 PMC210157

[22] Hook-Barnard IG, Hinton DM. Transcription initiation by mix and match elements: flexibility for polymerase binding to bacterial promoters. Gene Regul Syst Bio. 2007;1:275–93. 19119427 PMC2613000

[23] Chen Q, Stibitz S. The BvgASR virulence regulon of Bordetella pertussis. Curr Opin Microbiol. 2019;47:74–81. 10.1016/j.mib.2019.01.002 30870653

[24] Chen Q, Boulanger A, Hinton DM, Stibitz S. Strong inhibition of fimbrial 3 subunit gene transcription by a novel downstream repressive element in Bordetella pertussis. Mol Microbiol. 2014. 10.1111/mmi.12690 PMC4669884 24963821

[25] Chen Q, Lee G, Craig C, Ng V, Carlson PE Jr., Hinton DM, et al A novel Bvg-repressed promoter causes vrg-like transcription of fim3 but does not result in the production of serotype 3 Fimbriae in the Bvg(-) mode Bordetella pertussis. J Bacteriol. 2018. 10.1128/JB.00175-18 PMC6153668 30061354

[26] Roy CR, Falkow S. Identification of Bordetella pertussis regulatory sequences required for transcriptional activation of the fhaB gene and autoregulation of the bvgAS operon. J Bacteriol. 1991;173(7):2385–92. 10.1128/jb.173.7.2385-2392.1991 2007557 PMC207791

[27] Baikalov I, Schroder I, Kaczor-Grzeskowiak M, Grzeskowiak K, Gunsalus RP, Dickerson RE. Structure of the Escherichia coli response regulator NarL. Biochemistry. 1996;35(34):11053–61. 10.1021/bi960919o 8780507

[28] Eldridge AM, Kang HS, Johnson E, Gunsalus R, Dahlquist FW. Effect of phosphorylation on the interdomain interaction of the response regulator, NarL. Biochemistry. 2002;41(51):15173–80. 10.1021/bi026254+ 12484754

[29] Diavatopoulos DA, Cummings CA, Schouls LM, Brinig MM, Relman DA, Mooi FR. Bordetella pertussis, the causative agent of whooping cough, evolved from a distinct, human-associated lineage of B. bronchiseptica. PLoS Pathog. 2005;1(4):e45 10.1371/journal.ppat.0010045 16389302 PMC1323478

[30] Stefanelli P, Mastrantonio P, Hausman SZ, Giuliano M, Burns DL. Molecular characterization of two Bordetella bronchiseptica strains isolated from children with coughs. J Clin Microbiol. 1997;35(6):1550–5. 9163480 10.1128/jcm.35.6.1550-1555.1997 PMC229785

[31] Stibitz S, Yang MS. Subcellular localization and immunological detection of proteins encoded by the vir locus of Bordetella pertussis. J Bacteriol. 1991;173(14):4288–96. 10.1128/jb.173.14.4288-4296.1991 2066330 PMC208088

[32] Stemmer WP, Morris SK. Enzymatic inverse PCR: a restriction site independent, single-fragment method for high-efficiency, site-directed mutagenesis. Biotechniques. 1992;13(2):214–20. 1327007

[33] Merkel TJ, Stibitz S. Identification of a locus required for the regulation of bvg-repressed genes in Bordetella pertussis. J Bacteriol. 1995;177(10):2727–36. 10.1128/jb.177.10.2727-2736.1995 7751282 PMC176943

[34] Stark MJ. Multicopy expression vectors carrying the lac repressor gene for regulated high-level expression of genes in Escherichia coli. Gene. 1987;51(2–3):255–67. 10.1016/0378-1119(87)90314-3 3110013

[35] Stibitz S. Use of conditionally counterselectable suicide vectors for allelic exchange. Methods Enzymol. 1994;235:458–65. 10.1016/0076-6879(94)35161-9 8057916

[36] Vanderpool CK, Armstrong SK. The Bordetella bhu locus is required for heme iron utilization. J Bacteriol. 2001;183(14):4278–87. 10.1128/JB.183.14.4278-4287.2001 11418569 PMC95318

[37] Miller JH. Experiments in Molecular Genetics. Cold Spring Harbor Laboratory Press, New York 1972.

[38] Brickman E, Beckwith J. Analysis of the regulation of Escherichia coli alkaline phosphatase synthesis using deletions and phi80 transducing phages. J Mol Biol. 1975;96(2):307–16. 10.1016/0022-2836(75)90350-2 1100846

[39] Simon R, Priefer U, Pühler A. A Broad Host Range Mobilization System for In Vivo Genetic Engineering: Transposon Mutagenesis in Gram Negative Bacteria. Nature Biotechnology. 1983;1:784–91.

[40] Kasuga T, Nakase Y, Ukishima K, Takatsu K. Studies on Haemophilus pertussis. V. Relation between the phase of bacilli and the progress of the whooping-cough. Kitasato Arch Exp Med. 1954;27(3):57–62. 13296323

[41] Stibitz S, Carbonetti NH. Hfr mapping of mutations in Bordetella pertussis that define a genetic locus involved in virulence gene regulation. J Bacteriol. 1994;176(23):7260–6. 10.1128/jb.176.23.7260-7266.1994 7961497 PMC197114

