# Supplementary figures and images for: Multiple weak interactions between BvgA~P and ptx promoter DNA strongly activate transcription of pertussis toxin genes in Bordetella pertussis
Source: PLoS Pathog. 2020 May 13;16(5):e1008500. doi: 10.1371/journal.ppat.1008500 (PMC7250471; doi:10.1371/journal.ppat.1008500)

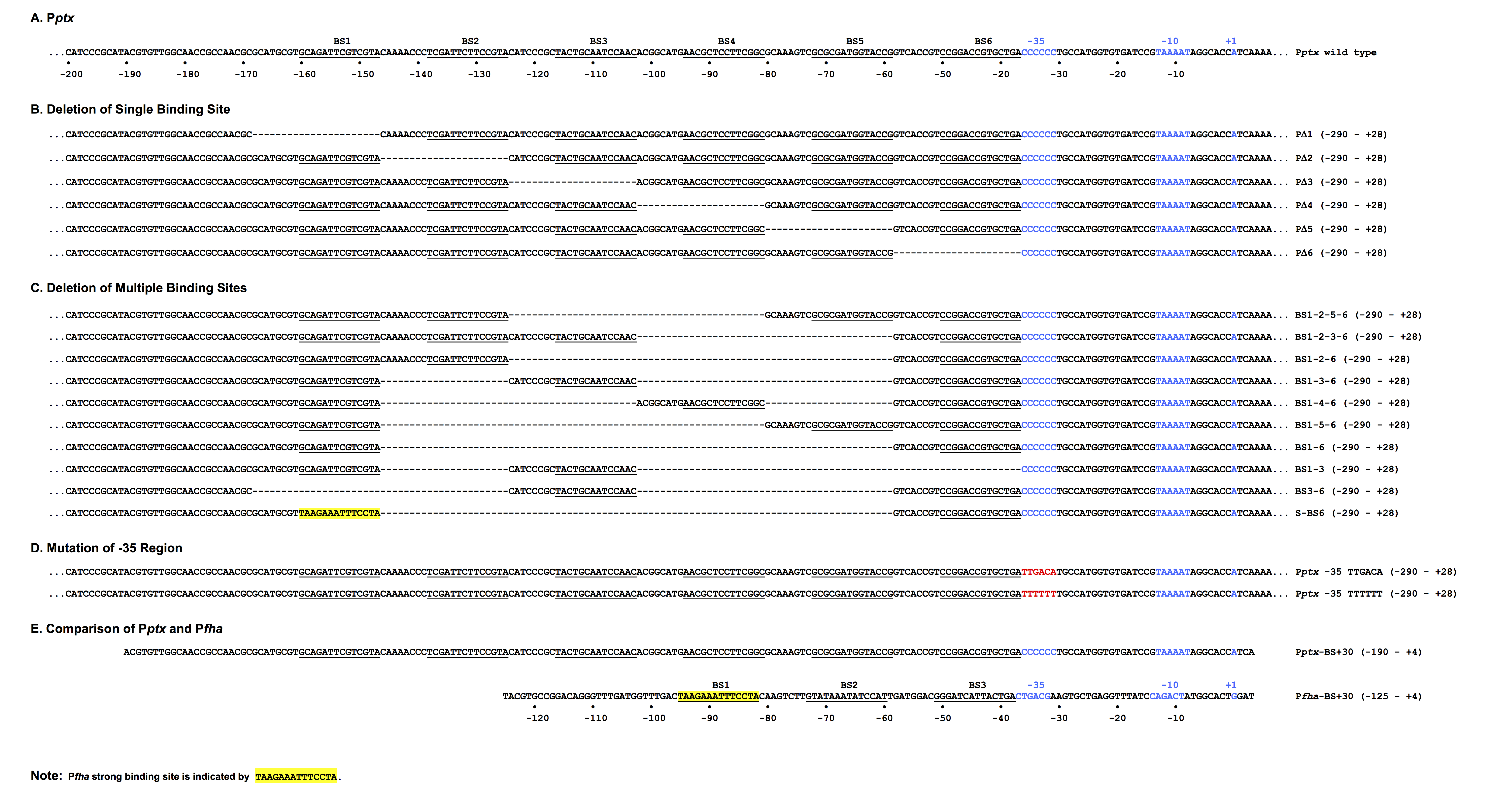

Supplement: S1 Fig — A. Wild-type Pptx. Binding sites for BvgA~P are underlined. Nucleotides in blue denote core promoter elements +1, -10 and -35. B & C. Deletion derivatives of Pptx with one (B) or more (C) BvgA binding sites deleted, as denoted by hyphens. In the variant S-BS6, yellow highlighting indicates the primary binding site from Pfha. D. Pptx variants with altered -35 regions, shown in red. E. Fragments used for comparisons of the strength of Pptx and Pfha. The Pfha strong binding site is highlighted. (TIF) [file ppat.1008500.s001.tif]
